# Understanding interoception across emotional contexts: development and validation of the Emotion-Linked Interoceptive Awareness Scale

**DOI:** 10.3389/fpsyg.2026.1757948

**Published:** 2026-03-18

**Authors:** Tomohiro Arai, Tomoko Komano, Hideki Ohira

**Affiliations:** 1 Shiseido Co., Ltd., MIRAI Technology Institute, Yokohama, Japan; 2 Graduate School of Informatics, Nagoya University, Nagoya, Japan; 3 Shiseido Co., Ltd., Brand Value R&D Institute, Yokohama, Japan

**Keywords:** bifactor model, body awareness, emotion, interoception, interoceptive attention, interoceptive sensibility, self-report measure

## Abstract

This research aimed to develop and validate the Emotion-Linked Interoceptive Awareness (ELIA) scale, a self-report measure assessing awareness of bodily sensations under specific emotional contexts, through four online studies. Study 1 generated items based on free-text reports of bodily sensations during specific emotions. Study 2 supported a bifactor structure comprising one general and three subgroup factors (namely, Positive Emotions, Anxiety, Irritation), thereby yielding a 39-item scale. The general factor reflected cross-emotion bodily awareness, with relatively stronger representation of positive emotional contexts. Study 3 demonstrated convergence with the Multidimensional Assessment of Interoceptive Awareness, particularly the Emotional Awareness subscale, and moderate test–retest reliability. Study 4 revealed that the ELIA Positive Emotions subscale scores were associated with lower trait anxiety and alexithymia, whereas the Anxiety and Irritation subscale scores were associated with higher levels of these traits. Mind–body practitioners scored higher on the ELIA, particularly on the Positive Emotions subscale, in matched comparisons. Mediation analyses indicated that the ELIA Positive Emotions subscale score mediated the associations of practitioner status with trait anxiety and with Externally Oriented Thinking, a facet of alexithymia, whereas the ELIA Total score mediated only the association with Externally Oriented Thinking. Overall, the ELIA provides an emotion-specific assessment that may help distinguish adaptive from maladaptive attentional styles of interoceptive sensibility and highlights the value of tracking bodily awareness within emotional contexts. More broadly, these findings suggest that developing and employing domain- and context-specific interoceptive self-report measures can yield deeper insights.

## 1 Introduction

### 1.1 Interoception and its conceptual subdivisions

In recent years, there has been growing interest in how bodily sensations are processed, perceived, appraised, and conceptually represented in the brain, and how they influence psychological functioning (Khalsa et al., 2018). This line of inquiry has led to the emergence of interoception as a key construct in understanding the relationship between bodily states and mental processes. Interoception is defined as the brain’s representation of internal bodily sensations (including visceral, thermal, and nociceptive information) transmitted through the humoral, lamina I spinothalamic, and vagal afferent pathways, contributing to the integrated perception of the body’s homeostatic state (Berntson and Khalsa, 2021; Craig, 2003).

Three conceptual subdivisions of interoception have been proposed, each with a distinct measurement approach (Garfinkel et al., 2015). First, interoceptive sensibility is assessed using self-report questionnaires that capture individuals’ perceived tendency to notice or attend to bodily sensations in daily life. Second, interoceptive accuracy is assessed by the performance on tasks that require tracking internal signals, such as heartbeat counting (Schandry, 1981) and discrimination (Whitehead et al., 1977). Finally, interoceptive awareness is assessed based on the correspondence between the individuals’ confidence in their task performance and their actual performance.

Building on this classification based on measurement methods (i.e., self-report versus performance-based), Murphy et al. (2019) proposed a second axis regarding the measurement target: accuracy versus attention. Accuracy determines how well one’s perception reflects actual bodily states, whereas attention refers to the degree to which bodily signals are the focus of conscious monitoring. For example, in self-report measures, accuracy is typically assessed using confidence ratings, whereas attention is reflected in beliefs about how frequently one attends to bodily sensations in everyday life.

Interoception research exhibits potential for advancing basic science and offering valuable insights into applied fields, including clinical domains such as mental health treatments (Khalsa et al., 2018; Solano Durán et al., 2024) and consumer applications such as the affective design of fragrances (Arai et al., 2024). Among the various facets of interoception, interoceptive sensibility, typically assessed through self-report questionnaires, offers a feasible and scalable approach compared with behavioral or physiological measures, which often require specialized equipment and technical expertise. This makes it useful in research and application settings.

### 1.2 Existing measures of interoceptive sensibility

To assess interoceptive sensibility, two self-report questionnaires have been frequently employed in psychological research: the Multidimensional Assessment of Interoceptive Awareness (MAIA; Mehling et al., 2012) and the Body Perception Questionnaire (BPQ; Porges, 1993), specifically, its Body Awareness subscale (BPQ-BA).

The MAIA was developed to capture potential changes resulting from mind–body practices (MBP) (such as mindfulness meditation, yoga, or tai chi) in the perception of bodily sensations and how these sensations are appraised, trusted, and regulated. It comprises eight subscales which reflect the multidimensional construct of bodily awareness relevant to clinical and mind–body therapeutic contexts: Noticing, Not-Distracting, Not-Worrying, Attention Regulation, Emotional Awareness, Self-Regulation, Body Listening, and Trusting. Previous studies have shown that MAIA scores correlate negatively with anxiety and alexithymia (Clemente et al., 2024; Trevisan et al., 2019) and that engagement in MBPs is associated with increased MAIA scores (Bornemann et al., 2015; Mehling et al., 2018).

By contrast, the BPQ, developed by Porges (1993) based on the polyvagal theory, is a broader self-report instrument assessing the perceived functioning and reactivity of autonomic nervous system-regulated organs. Among its subscales, the Body Awareness and Autonomic Reactivity subscales have received the most empirical attention and are the most used in research (Cabrera et al., 2018). The BPQ-BA items describe bodily sensations with well-established neuroanatomical bases (e.g., the urge to swallow, which is mediated by the vagus and glossopharyngeal nerves). BPQ-BA scores have been shown to correlate positively with somatosensory amplification (i.e., the tendency to experience benign bodily sensations as intense, disturbing, and salient) (Cabrera et al., 2018; Wang et al., 2020), anxiety, and alexithymia (Clemente et al., 2024; Trevisan et al., 2019).

Although the two measures are often assumed to assess similar aspects of an individual’s self-reported interoceptive profile, they are increasingly regarded as reflecting distinct attentional styles. Particularly, the attention style is adaptive in the case of the MAIA but maladaptive in the case of the BPQ-BA (Mehling, 2016; Trevisan et al., 2021, 2023). While the MAIA emphasizes a mindful, accepting, and self-regulatory attentional engagement with bodily sensations, the BPQ-BA is believed to reflect a hypervigilant attentional monitoring of bodily sensations, often driven by anxiety or physical discomfort. Importantly, however, the MAIA was originally designed to capture both the adaptive and maladaptive aspects of body awareness (Mehling et al., 2012). This intention is reflected in the Not-Distracting and Not-Worrying subscales, which are intended to capture maladaptive tendencies, such as the inclination to distract oneself from or to worry about bodily sensations.

These differences in conceptual focus and trait correlates underscore the importance of viewing interoceptive sensibility not as a unitary construct but rather as a shared domain that manifests through multiple, qualitatively distinct attentional modes.

### 1.3 Challenges of current interoceptive sensibility measures

While differences in attentional styles captured by interoceptive sensibility measures have been acknowledged, recent studies have raised concerns that various interoceptive sensibility questionnaires and even subscales within the same instrument may not assess the same underlying construct. This challenges the assumption that these tools are interchangeable or that they collectively measure a unified aspect of interoceptive sensibility.

Desmedt et al. (2022) conducted correlation, factor, and network analyses on five of the most used self-report scales for interoceptive sensibility, including the BPQ and MAIA. Their results revealed only weak-to-moderate inter-scale correlations (*r* < 0.60). Further, items from each scale were typically loaded onto distinct factors or formed separate communities in the network analysis. Thus, the foregoing suggests a limited conceptual overlap between the self-report scales. Specifically, the BPQ and MAIA items consistently formed independent clusters across all analytical approaches. Consistent with these findings, Vig et al. (2022) reported similarly weak-to-moderate correlations (Spearman’s *ρ* < 0.50), further underscoring the lack of convergence among widely used interoceptive sensibility scales.

Further complicating the landscape, Desmedt et al. (2022) reported that the MAIA subscales Not-Distracting and Not-Worrying were consistently loaded onto separate factors and formed distinct network communities, whereas the remaining six subscales displayed a more unified structure. This internal divergence within the MAIA was independently corroborated by Ferentzi et al. (2021), who conducted a confirmatory bifactor analysis on MAIA items. It was found that the Not-Distracting and Not-Worrying subscales contributed substantially less to the general factor (intended to represent a unified construct of interoceptive sensibility) than the other subscales. Collectively, these findings challenge the assumption that the MAIA uniformly captures a single coherent construct, underscoring the lack of conceptual convergence, even within a single instrument.

These discrepancies underscore the need for a more nuanced understanding of interoceptive sensibility. However, it is unclear whether a unified concept of interoceptive sensibility exists. Nonetheless, developing and employing domain-specific measures alongside comprehensive tools such as MAIA may offer deeper insights into the complex nature of interoception (Clemente et al., 2024).

### 1.4 Theoretical rationale for emotion-specific interoceptive assessment

While differences in attentional style (such as adaptive versus maladaptive modes) have been discussed in interoceptive sensibility research, it remains unclear whether these distinctions apply uniformly across various subdomains such as Noticing or Emotional Awareness. This uncertainty is heightened by recent findings suggesting that these subdomains may not converge on a single underlying construct. Given the growing evidence that interoception and emotion are closely intertwined, emotion-linked bodily awareness is a promising focus for extending the attentional style framework.

Recent reviews have emphasized the foundational role of bodily signals in generating, regulating, and experiencing emotion (Barrett, 2017; Critchley and Garfinkel, 2017; Greenwood and Garfinkel, 2025). This emphasis is consistent with classic emotion theories that highlight interoceptive contributions (Damasio, 1996; James, 1884; Schachter and Singer, 1962). Despite the expanding literature, influential reviews in this field have largely concentrated on negative emotions such as fear, disgust, and anxiety (Critchley and Garfinkel, 2017; Greenwood and Garfinkel, 2025). The relationship between positive emotions and bodily awareness remains understudied. Accordingly, interoception studies of negative emotions and related disorders have primarily informed the development and refinement of clinical interventions. The clinical implications of positive emotions remain less well characterized.

Regarding interoceptive sensibility, the MAIA includes a subscale titled Emotional Awareness, which reflects the link between bodily sensations and emotions. This subscale’s inclusion suggests that, even in self-report measures of interoception, the interoception–emotion link is a key component. However, the subscale comprises only five broadly worded items (e.g., “I notice my body feels different after a peaceful experience”), limiting its ability to capture the diversity of emotion-specific bodily experiences. It does not distinguish between positive and negative emotional contexts or adaptive and maladaptive interoceptive styles. This limitation is consistent with the evidence that Emotional Awareness is not reliably associated with trait anxiety, as reported by Mehling et al. (2012) and in a recent comprehensive review (Clemente et al., 2024).

This limitation calls for considering whether interoceptive attention’s adaptiveness varies by emotional context. In positive emotional states, bodily awareness may serve adaptive functions, though direct evidence for the adaptiveness of bodily awareness within positive emotional contexts is scarce. Nevertheless, indirectly relevant work has indicated that MBPs, frequently discussed alongside MAIA-assessed interoceptive sensibility, may foster positive emotion and adaptive behavior. For example, MBPs have been demonstrated to increase awareness of positive experiences in daily life (Lindsay et al., 2018). Further, Mindfulness-to-Meaning Theory posits that mindfulness fosters positive reappraisal and biases attention toward pleasant events, thereby heightening awareness of rewarding experiences, increasing positive emotion during everyday activities, and, in turn, reinforcing positive reappraisal (Garland et al., 2015). By contrast, in negative emotional states, bodily awareness may co-occur with symptom-focused attention, including somatic hypervigilance and somatosensory amplification (frequently discussed in relation to BPQ-BA-assessed interoceptive sensibility). However, direct evidence highlighting that bodily awareness is maladaptive within negative emotional contexts remains limited. Nevertheless, findings linking negative affect to symptom reporting provide indirect support for this possibility: Higher negative affect is associated with greater symptom reporting and more severe pain among patients with chronic pain (O’Brien et al., 2008), and experimentally induced negative affect heightens self-reported physical symptoms among patients with functional somatic syndromes (Bogaerts et al., 2023). Collectively, these findings suggest that interoceptive attention’s adaptiveness depends on the emotional context in which it occurs.

In sum, these considerations highlight the need for a dedicated measure of emotion-linked interoceptive sensibility that explicitly distinguishes between adaptive and maladaptive attentional styles within emotionally salient contexts. Such a measure would offer a more nuanced framework for understanding how bodily awareness contributes to emotional functioning and psychological health.

### 1.5 The present study

This study develops and validates a novel self-report measure of interoceptive sensibility, focusing on bodily awareness across emotional contexts. This scale primarily aims to assess the Emotional Awareness sub-dimension of interoceptive sensibility with greater resolution. While retaining the core construct of Emotional Awareness, the scale offers enhanced granularity in characterizing both the emotional situations that elicit bodily sensations and the qualitative features of those sensations. As a secondary contribution, by situating bodily awareness within clearly defined emotional contexts, the scale provides an opportunity to discuss adaptive and maladaptive interoceptive attentional styles within the Emotional Awareness sub-dimension. Together, these contributions help address the existing literature’s aforementioned limitations. Additionally, the scale is designed to be comprehensible and easy to complete for members of the general population, avoiding the abstraction and technical demands of some existing measures (Mehling et al., 2012). Overall, these characteristics constitute the scale’s incremental contribution to understanding the Emotional Awareness sub-dimension of interoceptive sensibility.

Scale development and validation were conducted through four studies. Study 1 generated candidate items from free-text responses in which participants described the bodily sensations they typically experienced during different emotional states. Study 2 identified the scale’s latent structure through exploratory and confirmatory factor analyses using a bifactor modeling approach. This approach tested whether all items loaded onto a single underlying construct (assuming a general construct spanning different emotions) while also accommodating distinct sub-dimensions associated with specific emotional contexts. Study 3 examined construct validity through correlations with existing measures (particularly the MAIA Emotional Awareness), evaluated test–retest reliability (based on the assumption that interoceptive sensibility is a stable, trait-like construct), and collected participants’ evaluations of the scale’s comprehensibility and ease of use. Study 4 assessed the scale’s ability to distinguish between attentional style modes by comparing it to the MAIA and BPQ-BA and by examining associations with interoception-related traits such as trait anxiety and alexithymia. This study also investigated whether the scale was sensitive to individual differences linked to long-term engagement in MBPs, which are theorized to promote adaptive interoceptive attention. All four studies were conducted with Japanese adults, potentially constraining the cross-cultural generalizability of the findings obtained with the scale; this limitation is discussed in the Discussion section. Together, these studies introduce a theoretically grounded, psychometrically sound, and practically useful instrument for assessing interoceptive sensibility in emotional contexts.

Based on theoretical accounts and prior findings, two primary hypotheses are tested. First, the scale is hypothesized to exhibit a bifactor structure, comprising a general factor representing bodily awareness across emotional contexts and distinct subfactors capturing bodily awareness specific to particular emotional contexts. Second, bodily awareness across different emotional contexts is expected to exhibit distinct patterns of association with established interoception measures and related psychological traits. Specifically, bodily awareness during positive emotions is expected to more strongly correlate with the MAIA and with favorable psychological and behavioral correlates, including lower trait anxiety, lower alexithymia, and greater engagement in MBPs. By contrast, bodily awareness during negative emotions is expected to more strongly correlate with the BPQ-BA and with less favorable psychological correlates, including higher trait anxiety and higher alexithymia. Collectively, these differential correlational profiles are expected to support the notion that interoceptive attentional styles vary by emotional context, with bodily awareness within positive emotional contexts reflecting a more adaptive style and bodily awareness within negative emotional contexts reflecting a more maladaptive style.

## 2 Method

### 2.1 Overview of studies

**Figure 1:**
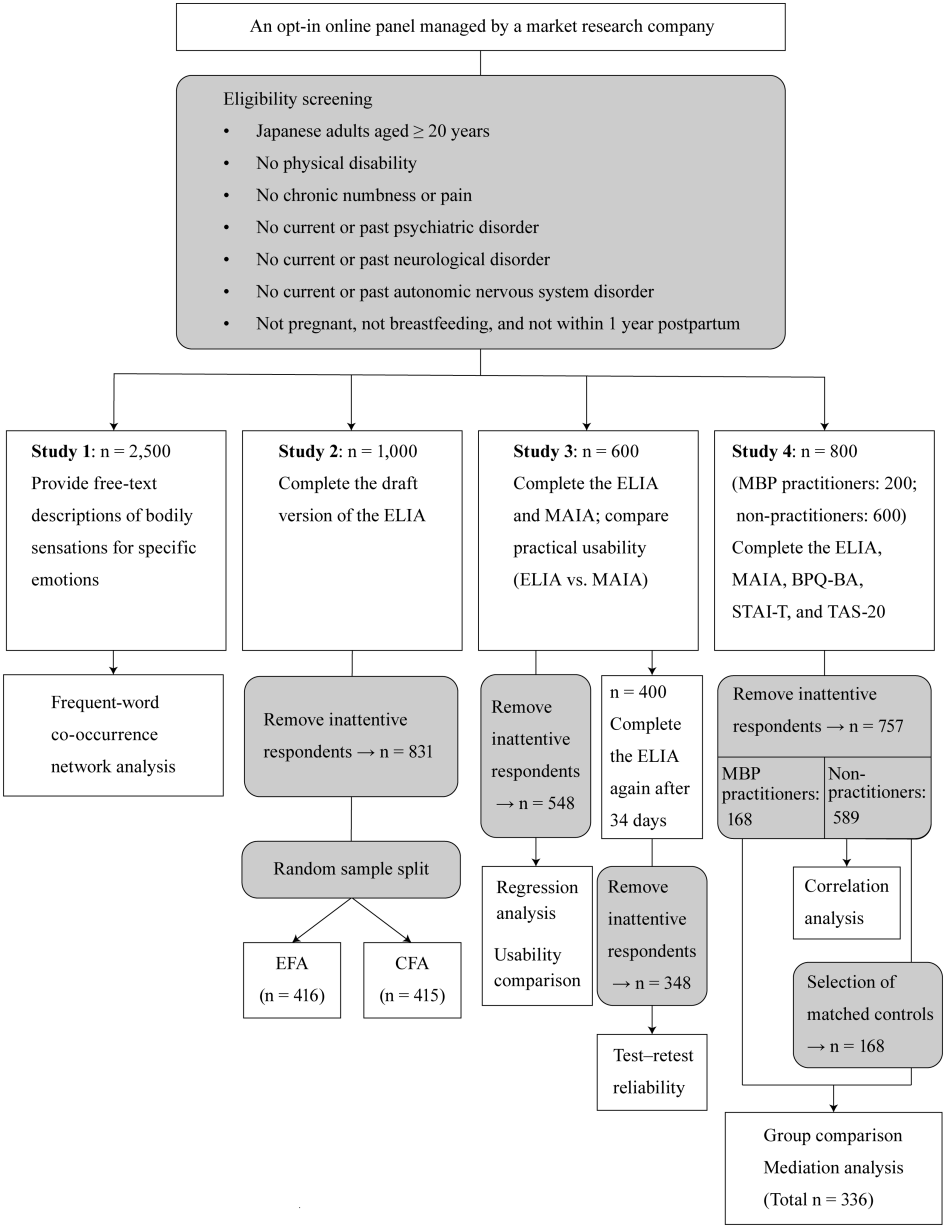
Flowchart of participant sampling, survey procedures, and data analyses across Studies 1–4. This research comprised four online surveys conducted sequentially from Studies 1 to 4, with no temporal overlap. The figure summarizes participant sampling and eligibility screening, assessment components, data cleaning procedures (specifically the exclusion of inattentive respondents identified by a trap item), group-matching procedures, and the data-analytic steps for each study. EFA, exploratory factor analysis; CFA, confirmatory factor analysis; ELIA, Emotion-Linked Interoceptive Awareness; MAIA, Multidimensional Assessment of Interoceptive Awareness; BPQ-BA, Body Perception Questionnaire–Body Awareness; STAI-T, State–Trait Anxiety Inventory, Trait; TAS-20, 20-item Toronto Alexithymia Scale; MBP, body–mind practice.

This research encompassed four sequential online studies. Figure 1 illustrates each study’s sampling and screening procedures, assessment components, and subsequent data-cleaning and analytic steps. All surveys were administered among Japanese adults, recruited from an opt-in online panel managed by a market research company (i.e., a non-probability sample). The eligibility criteria were as follows: (a) age ≥ 20 years; (b) no physical disability; (c) no chronic numbness or pain; (d) no current or past psychiatric disorder; (e) no current or past neurological disorder; (f) no current or past autonomic nervous system disorder; and (g) not pregnant, not breastfeeding, and not within 1 year postpartum. Except for criterion (a), these criteria were intended to minimize physiological and clinical factors that could influence bodily sensations and their perception. Potential limitations associated with non-probability sampling using an online panel and the restriction to non-pregnant, non-clinical Japanese adults without physical disabilities are detailed in the Discussion.

Study 1 collected and analyzed individuals’ free-text descriptions of the bodily sensations experienced under specific emotional states to inform item development for the ELIA scale. In Study 2, exploratory and confirmatory factor analyses were performed to determine the latent factor structure of the ELIA scale. Study 3 evaluated whether the ELIA scale was predominantly associated with the Emotional Awareness subscale of the MAIA. The ease of understanding and responding to the ELIA scale was compared to that of the MAIA, and the test–retest reliability of the ELIA scale was examined. Study 4 investigated the associations of the new scale with both the MAIA and BPQ-BA, as well as its relationships with trait anxiety and alexithymia. ELIA scale scores were also compared between individuals with and without MBP engagement. Additionally, mediation analyses were conducted to examine whether ELIA scale scores mediated the relationships between MBP engagement and both trait anxiety and alexithymia. All studies were approved by the Ethics Committee of Shiseido Global Innovation Center. Informed consent was obtained from all participants. All statistical analyses were performed using R version 4.3.3 (R Core Team, 2024), with the significance level (*α*) set at 0.05.

### 2.2 Item development (Study 1)

To develop items for the new ELIA scale, free-text descriptions of emotion-linked bodily sensations were collected. A total of 2,500 Japanese adults (1,250 women and 1,250 men) participated in this study. Their ages ranged from 20 to 69 years [mean age = 44.61 years, standard deviation (SD) = 13.86 years]. Participants were asked to freely describe the bodily sensations they typically experienced when each of the following eight emotions was evoked: happiness, excitement, relaxation, contentment, anxiety, irritation, sadness, and boredom. These emotional states were selected following the Circumplex Model of Emotions (Russell, 1980), which organizes perceived emotions within a two-dimensional valence-arousal space. Two emotions were selected from each circumplex quadrant to ensure coverage across emotions that differed in valence and arousal combinations. Moreover, we selected emotion labels that can be reported during periods of physiological changes in everyday life (Hoemann et al., 2020). A co-occurrence network analysis was performed to identify verbal expressions (i.e., keywords used in combination) that were frequently and commonly reported to describe the bodily sensations associated with each emotional state. Based on the results of this analysis, 66 items were generated for use in subsequent studies (Supplementary materials, Section 1 [“Item Development Based on Free-Text Data (Study 1)”], provides details of the co-occurrence network analysis).

### 2.3 Exploratory and confirmatory factor analyses (Study 2)

#### 2.3.1 Participants and procedure

To collect data for exploratory and confirmatory factor analyses, 1,000 Japanese adults (500 women and 500 men) were asked to complete the 66 items developed in Study 1. Their ages ranged from 20 to 69 years (mean age = 44.78 years, SD = 14.03 years). The participants were asked to indicate how often they experienced the bodily sensations described by each item when the emotion specified by that item was evoked. Each item was rated on a scale ranging from 0 (never) to 5 (always). Items associated with each of the eight emotions were grouped into blocks to reduce the cognitive burden of completing the items. The presentation order of the items within each block as well as that of the blocks themselves was counterbalanced across participants. To identify inattentive respondents, a trap item was embedded among the items (Gao et al., 2016), instructing participants to select a specific response option (e.g., “Please choose the second number from the right as your answer.”). Participants who failed to respond correctly to the trap items were excluded from the analysis.

#### 2.3.2 Exploratory bifactor analysis

An exploratory factor analysis (EFA) was performed on half of the available datasets to identify the latent structure of the 66 items. Before the EFA, data adequacy was evaluated using the Kaiser–Meyer–Olkin procedure, which yielded the overall measure of sampling adequacy (MSA) and item-level MSAs derived from the anti-image correlation matrix. After confirming the adequacy, the number of lower-order factors to be retained was automatically determined using parallel analysis.

A Direct Schmid–Leiman (DSL) procedure was then applied to obtain an exploratory bifactor solution. DSL enables exploratory bifactor analysis from a single first-level factor analysis by orthogonally Procrustes-rotating an augmented loading matrix to a bifactor target derived from an obliquely rotated solution, yielding orthogonal general and subgroup factors; no separate higher-order analysis is required (Waller, 2018). Based on simulation evidence, the DSL method is recommended for small to moderately sized samples (e.g., *N* = 200–500) when estimating exploratory hierarchical or non-hierarchical bifactor models, as it is among the most accurate relative to alternatives (Giordano and Waller, 2020).

An unrotated k-factor solution was first extracted using maximum likelihood. The solution was then obliquely rotated with oblimin to achieve a simple lower-order structure, from which a signed dichotomized bifactor target matrix was constructed. Finally, Schmid–Leiman loadings were obtained by orthogonal Procrustes rotation of the augmented first-level solution to this target, yielding orthogonal general and group factors. The resulting loadings matrix corresponds to a rank-deficient (hierarchical) Schmid–Leiman representation. All the steps were performed in R using the BiFAD function in the fungible package (Waller, 2024). Items were excluded in any of the following cases: (i) the absolute loading on the general factor was ≤ 0.45; (ii) all subgroup-factor loadings were ≤ 0.45 in absolute value; or (iii) absolute cross-loadings > 0.30 were observed on more than one subgroup factor (Comrey and Lee, 1992). Item exclusion was conducted to retain the general factor while achieving a simple structure for the subgroup factors. Specifically, each item was expected to load on the general factor and on only one subfactor, thereby enhancing the factor structure’s interpretability. The EFA–DSL cycle was repeated on the retained items until a simple factor structure was obtained.

#### 2.3.3 Confirmatory bifactor analysis

A confirmatory factor analysis (CFA) was conducted using the remaining half of the datasets to validate the factor structure identified in the exploratory bifactor analysis. The model was specified based on the results of the exploratory analysis. The subgroup factors were initially constrained to be orthogonal (i.e., correlations fixed at zero). Subsequently, inter-subgroup correlations with modification indices > 10.83 were freely estimated to enhance model parsimony and interpretability. Model parameters were estimated using robust maximum likelihood (Yuan and Bentler, 2000). Model fit was evaluated using the Comparative Fit Index (CFI), Root Mean Square Error of Approximation (RMSEA), and Standardized Root Mean Square Residual (SRMR). Following established guidelines, CFI > 0.90, RMSEA < 0.08, and SRMR < 0.08 were considered indicators of acceptable fit (Hu and Bentler, 1999). Moreover, the 90% confidence interval (CI) for the RMSEA was computed. In addition to the bifactor model, alternative models were tested for comparison purposes: a unidimensional model, a correlated-factor model, and a hierarchical (second-order) model. To further assess internal consistency, explained common variance (ECV), omega hierarchical (ωH), and omega hierarchical subscale (ωHS) were calculated (Rodriguez et al., 2016). ECV and ωH assess the extent to which a general factor explains variance across items, supporting the assumption of unidimensionality. Conversely, ωHS evaluates the reliability of each subscale after accounting for the general factor, indicating the degree of unique variance that each subscale captures. According to recommended thresholds, acceptable reliability is defined as ECV > 0.60 and ωH > 0.70 for the general factor, and ωHS > 0.50 for the subscales (Grygiel et al., 2016). These analyses were conducted in R using the lavaan package (Rosseel, 2012) and BifactorIndicesCalculator package (Dueber, 2025).

### 2.4 Validation of emotional specificity and reliability (Study 3)

#### 2.4.1 Participants and procedure

To examine whether the newly developed ELIA scale primarily captures awareness of bodily sensations specific to emotional contexts and demonstrates adequate test–retest reliability, its relationship with the Emotional Awareness subscale of the MAIA was assessed, and the consistency of scores across two different time points was evaluated. A total of 600 Japanese adults (300 women and 300 men) completed both the ELIA scale and the Japanese version of the MAIA (Shoji et al., 2018), with the presentation order of the two scales randomized across participants. Their ages ranged from 20 to 69 years (mean age = 44.60 years, SD = 14.00 years). To assess test–retest reliability, a subset of 400 participants (195 women and 205 men, mean age = 46.24 years, SD = 13.78 years) completed the ELIA scale again after 34 days. Trap items were embedded in both the initial and follow-up administrations of ELIA and MAIA to identify inattentive respondents. Participants who failed to answer either item correctly were excluded from subsequent analyses. Additionally, all 600 participants were asked to rate the ease of understanding and responding to the ELIA scale compared with the MAIA scale.

#### 2.4.2 Associations with MAIA

To evaluate the construct validity of the ELIA scale, its association with subscales of the Japanese version of the MAIA was assessed. The Japanese MAIA consists of 25 items and six subscales: Attention Regulation, Body Listening, Noticing, Emotional Awareness, Trusting, and Not-Distracting (Shoji et al., 2018). These subscales differ from the original eight-factor structure (Mehling et al., 2012) because Shoji et al. did not retain the Self-Regulation and Not-Worrying subscales in their Japanese adaptation. The remaining subscales retained their original labels, although the Body Listening, Noticing, and Emotional Awareness subscales consisted of slightly different item sets from their original counterparts. Each item was rated on a 6-point scale ranging from 0 (never) to 5 (always). Higher subscale scores reflect greater interoceptive sensibility. All subscales demonstrated acceptable internal consistency (McDonald’s ω_t_ = 0.71–0.97). The ELIA scale also showed excellent internal consistency for both its total score and three subscales (McDonald’s ω_t_ = 0.93–0.98).

To assess the strength and specificity of the associations between the MAIA sub-dimensions and the ELIA scale, a multiple linear regression analysis was conducted using the mean of the 39 ELIA items (hereafter referred to as the total score) as the dependent variable and the six MAIA subscales (each computed as the mean of its items) as independent variables. All independent and dependent variables were standardized using z-transformation before entering them into the model. Multicollinearity was assessed using variance inflation factors (VIF). The overall model fit was evaluated using an F-test comparing the full model to an intercept-only model. As a general guideline for interpreting standardized regression coefficients, values of |*β*| ≈ 0.10 are considered small, |*β*| ≈ 0.30 moderate, and |*β*| ≥ 0.50 large in magnitude (Cohen, 1988).

#### 2.4.3 Ease of use comparison

To compare the perceived clarity and ease of responding to the items between the ELIA and the MAIA, participants were asked, after completing both scales, to indicate which set of items they found easier to understand and answer. They responded to a forced-choice question: which was easier to comprehend and respond to, the ELIA scale or the MAIA. The responses were coded as binary variables (ELIA = 1, MAIA = 0). A binomial test was then conducted to determine whether the proportion favoring the ELIA scale differed significantly from chance (i.e., 0.50), thereby assessing whether one scale was consistently perceived as clearer and easier to answer.

#### 2.4.4 Test–retest reliability

To evaluate the temporal stability of the responses to the ELIA scale, test–retest reliability was assessed over a 34-day interval. Intraclass correlation coefficients (ICC) were calculated to estimate the consistency of the participants’ scores across the two administrations. Specifically, ICCs were computed for the total score (mean of all 39 items) and for each of the three subscale scores (mean of the items within each subscale). A two-way random-effects model with single measures and absolute agreement was used to quantify the score stability over time. ICCs were computed using the irr package (Gamer et al., 2019) in R. According to conventional guidelines, ICC values of 0.50 or higher were considered to indicate acceptable reliability (Koo and Li, 2016). 95% CIs were also computed for the ICC estimates.

### 2.5 Associations with interoceptive measures, related traits, and mind–body practices (Study 4)

#### 2.5.1 Participants and procedure

To further validate the ELIA scale, Study 4 examined its associations with established measures of interoceptive sensibility, interoception-related psychological traits, and long-term engagement in MBPs. A total of 800 Japanese adults (367 women and 433 men) aged 20–69 years (M = 49.48, SD = 12.05) completed the ELIA scale along with the Japanese versions of the MAIA, BPQ-BA (Kobayashi et al., 2021), State–Trait Anxiety Inventory–Trait Version (STAI-T; Hidano et al., 2000), and Toronto Alexithymia Scale (TAS-20; Komaki et al., 2003). The order of the scale presentation was randomized across participants. Additionally, they reported whether they engaged in regular MBPs, including yoga, mindfulness meditation, zazen, breathing meditation, body scan, tai chi, Pilates, or autogenic training, and specified the total duration of practice. Individuals reporting at least 5 years of MBP engagement were categorized as long-term practitioners (*n* = 200; 117 women and 83 men; age range = 24–69 years, *M* = 50.16, SD = 11.68), while those reporting no prior experience with any MBP were categorized as non-practitioners (*n* = 600; 250 women and 350 men; age range = 20–69 years, *M* = 49.26, SD = 12.17). To identify inattentive respondents, one trap item was embedded in each of the ELIA, MAIA, and BPQ-BA. Participants who failed to correctly answer any of these trap items were excluded from subsequent analyses.

#### 2.5.2 Associations with MAIA, BPQ-BA, STAI-T, and TAS-20

To examine how the ELIA scale relates to other self-report measures of interoceptive sensibility and relevant psychological traits, Pearson correlation analyses were conducted using data from non-practitioners only to avoid the potential confounding effects of long-term MBP. The ELIA total score, its three subscale scores (computed as item means), and the MAIA, BPQ-BA, STAI-T, and TAS-20 scores were analyzed.

The ELIA scale demonstrated excellent internal consistency across both total and subscale scores (McDonald’s ω_t_ = 0.93–0.98). For details of the Japanese MAIA, see Study 3; internal consistency was acceptable (McDonald’s ω_t_ = 0.65–0.97).

The Japanese very short form of the BPQ-BA (Kobayashi et al., 2021) was used, comprising 12 items rated on a 5-point scale from 1 (none) to 5 (always). Scores were averaged across items, with higher values indicating greater awareness of bodily sensations (McDonald’s ω_t_ = 0.91).

The Japanese version of the STAI-T (Hidano et al., 2000) includes 20 items rated on a 4-point scale from 1 (Almost Never) to 4 (Almost Always). After reverse-scoring the negatively worded items, responses to the 20 items were summed, with higher scores indicating greater trait anxiety (McDonald’s ω_t_ = 0.93).

The Japanese TAS-20 (Komaki et al., 2003) consists of 20 items in three subscales: Difficulty Identifying Feelings, Difficulty Describing Feelings, and Externally Oriented Thinking. Items were rated on a 5-point scale from 1 (Strongly Disagree) to 5 (Strongly Agree), and the total and subscale scores were computed as item means. Higher scores reflect greater alexithymic tendencies (McDonald’s ω_t_ = 0.73–0.89).

Pearson correlation coefficients were calculated, and two-tailed tests assessed whether each correlation significantly differed from zero. Within-instrument intercorrelations for ELIA and MAIA and correlations between the STAI-T and the TAS-20 were not examined because these analyses were not central to this study’s objectives. To correct for multiple comparisons, *p*-values were adjusted using the Benjamini–Hochberg false discovery rate (FDR) procedure (Benjamini and Hochberg, 1995). 95% CIs were also calculated for the correlation coefficients.

#### 2.5.3 Comparisons between MBP practitioners and non-practitioners

To examine how long-term engagement in MBPs relates to ELIA scale scores, group comparisons were conducted between MBP practitioners and non-practitioners using a matched-sample approach. To minimize the potential confounding effects of age and gender, propensity score matching was conducted using the MatchIt package (Ho et al., 2011) in R. Nearest-neighbor matching without replacement was performed based on the logistic regression-derived propensity scores using a 1:1 ratio. The matching diagnostics and outcomes are presented in Supplementary Figure 2.

After matching, independent-samples Welch’s t-tests were performed to compare the ELIA total and subscale scores between the practitioners and matched controls. Effect sizes were calculated using Cohen’s d, with values interpreted according to standard conventions (0.2 = small, 0.5 = medium, 0.8 = large; Cohen, 1988). To adjust for multiple comparisons across the ELIA total and subscale scores, p-values were corrected using the FDR method.

As supplementary analyses, group differences in the MAIA and BPQ-BA scores were also tested. However, because these comparisons were not central to the primary research aims, the results are reported in Supplementary Table 6.

Finally, group differences in trait anxiety (STAI-T) and alexithymia (TAS-20 total and subscales) were tested to confirm the expected psychological distinctions between MBP practitioners and controls. These comparisons were intended to establish a basis for the subsequent mediation analyses. As with the ELIA scores, the *p*-values were adjusted using FDR correction to account for multiple comparisons.

#### 2.5.4 Mediation analyses

Mediation analyses were conducted to examine whether interoceptive sensibility, as measured by the ELIA scale, statistically mediates the group differences in trait anxiety and alexithymia between long-term MBP practitioners and non-practitioners. The models were theory-driven and consistent with prior research wherein interoceptive sensibility was modeled as a mediator of mindfulness/MBP intervention or practice effects on psychological outcomes (de Jong et al., 2016; Fissler et al., 2016). These analyses were restricted to variables that exhibited significant group differences after FDR correction, drawn from ELIA (total and subscale scores), STAI-T, and TAS-20 (total and subscale scores).

Mediation models were tested to determine whether ELIA scores mediated the relationship between the MBP group (dummy coded: 1 = practitioner, 0 = control) and psychological trait outcomes. Analyses were conducted in R using the lavaan package, with observed-variable path models fit within a structural equation modeling (SEM) framework and estimated by maximum likelihood. Indirect effects were estimated using non-parametric bootstrapping with 5,000 resamples, and significance was evaluated based on 95% bias-corrected and accelerated confidence intervals (BCa CIs). Mediation was considered significant if the BCa CI for the indirect effect did not include a zero.

## 3 Results

### 3.1 Factor structure of the ELIA scale (Study 2)

One hundred sixty-nine participants (16.9%) failed to answer one or more trap items correctly and were excluded from the analysis, resulting in a final sample size of 831 participants (437 women and 394 men; mean age = 45.64 years, SD = 13.94 years).

Preliminary data screening confirmed the dataset’s suitability for factor analysis. In the inter-item correlation matrix, no pairwise absolute Pearson correlation coefficient exceeded 0.85 or fell below 0.22. The overall MSA was 0.97, with all individual MSA values exceeding 0.95.

Exploratory bifactor analysis using DSL transformation was conducted on the first half of the sample (*n* = 416). A bifactor structure consisting of one general factor and three specific subgroup factors was identified through four iterative refinements. These subgroup factors represent the awareness of bodily sensations during (1) positive emotions (including happiness, excitement, relaxation, and contentment), (2) anxiety, and (3) irritation. A total of 27 items, including all those reflecting bodily sensations during sadness and boredom, were excluded because of insufficient loadings or substantial cross-loading. The final structure consisted of 39 items. To illustrate the content of the retained items, three representative items from each of the three subgroup factors are listed in Table 1. A complete list of all items and their factor loadings is provided in Supplementary Table 1.

**Table 1:** Examples of ELIA items.

Subscale 1 (bodily sensations during positive emotions)
When I feel happy, I notice my body feeling light.
When I feel excited, I notice my body temperature rising and warmth spreading through my whole body.
When I feel content, I notice my breathing becoming slower, deeper, and easier.
Subscale 2 (bodily sensations during anxiety)
When I feel anxious, I notice my breathing becoming shallow.
When I feel anxious, I notice my palms becoming sweaty.
When I feel anxious, I notice my mouth and throat becoming dry.
Subscale 3 (bodily sensations during irritation)
When I feel irritated, I notice a rush of blood to my head.
When I feel irritated, I notice my heart beating faster.
When I feel irritated, I notice my muscles tensing up.

ELIA, Emotion-Linked Interoceptive Awareness. Supplementary Table 1 presents the full set of items.

**Figure 2:**
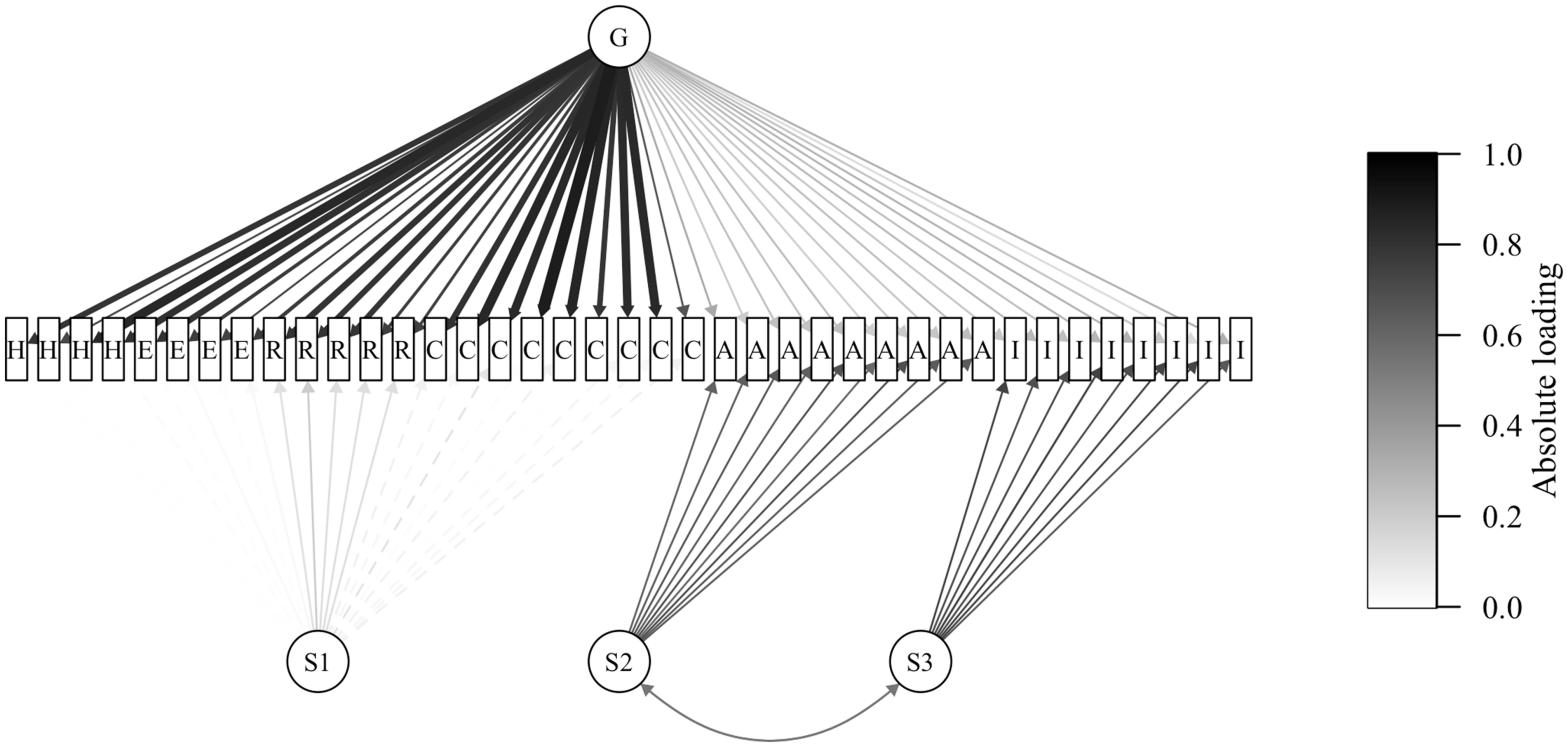
Schematic diagram of the CFA model for ELIA. *N* = 415; CFA, confirmatory factor analysis; ELIA, Emotion-Linked Interoceptive Awareness; G, general factor; S1–S3, subgroup factors; H, happiness; E, excitement; R, relaxation; C, contentment; A, anxiety; I, irritation. A bifactor model with one general factor (G) and three subgroup factors (S1–S3) was estimated using robust maximum likelihood. Each latent factor’s total variance was fixed at 1.0. Standardized loadings are presented; line thickness and shading scale with the absolute loading magnitude, while line type indicates the sign (solid = positive, dashed = negative). The G factor loads on all items, and each S factor (S1–S3) loads only on its designated items; cross-loadings other than the G factor were constrained to zero. The S2–S3 correlation was freely estimated, while all other inter-factor correlations were fixed at zero. Rectangles denote observed variables, and circles denote latent variables. Observed-variable labels indicate only the emotion category (H, E, R, C, A, I) and omit item numbers. Residuals are omitted for clarity. Supplementary Table 1 presents the full parameter estimates.

Confirmatory bifactor analysis was conducted on the second half of the sample (*n* = 415) supporting the identified structure, yielding acceptable fit indices: CFI = 0.919, RMSEA = 0.067 [90% CI (0.063, 0.072)], and SRMR = 0.037. A schematic diagram of the model is presented in Figure 2, and detailed loadings and model comparisons are provided in Supplementary Tables 1, 2. A model comparison confirmed that the bifactor model outperformed the unidimensional, correlated-factor, and second-order models.

Regarding internal consistency, the general factor demonstrated acceptable reliability (ECV = 0.65, ωH = 0.88). Reliability for the three subgroup factors after accounting for the general factor was as follows: ωHS values were < 0.01 for Positive Emotions, 0.69 for Anxiety, and 0.70 for Irritation. The Positive Emotions subscale had limited unique variance beyond the general factor.

Considering the minimal unique variance of the Positive Emotions subgroup factor beyond the general factor, a sensitivity analysis was conducted to evaluate whether the overall bifactor structure depended on this subgroup factor. Model fit and bifactor indices were compared between the primary model and an alternative specification wherein the Positive Emotions items loaded solely on the general factor. Fit indices and bifactor indices were largely comparable across the two models, though the CFI decreased to 0.891 (Supplementary Table 3).

### 3.2 Construct validity and test–retest reliability of the ELIA scale (Study 3)

Fifty-two participants (8.7%) failed to answer one or more trap items correctly and were excluded from the analysis, resulting in a final sample size of 548 participants (284 women and 264 men; mean age = 45.04 years, SD = 13.78 years).

Construct validity was examined by testing the association between ELIA scores and the sub-dimensions of interoceptive sensibility as measured by the Japanese MAIA. Multiple linear regression analysis was conducted with the ELIA total score as the dependent variable and the six MAIA subscales as independent variables. The overall model was significant, *F*(6, 541) = 72.61, *p* < 0.001, with an explained variance (R^2^) of 0.45. VIFs for all independent variables were below 10, indicating no serious multicollinearity.

Table 2 summarizes the standardized regression coefficients (*β*), their 95% CIs, t-values, and *p*-values for each MAIA subscale. Statistically significant positive associations with the ELIA scores were observed for Emotional Awareness and Noticing, whereas Not-Distracting showed a significant negative association. Among these factors, Emotional Awareness exhibited the strongest association. The remaining subscales were not significantly associated with the ELIA scores.

**Table 2:** Contributions of MAIA subscales to prediction of ELIA total score.

Variables	*β*	95% CI	*t*	df	*p*
Attention Regulation	−0.10	[−0.27, 0.07]	−1.19	541	0.234
Body Listening	0.11	[−0.04, 0.26]	1.50	541	0.135
Noticing	0.22	[0.08, 0.35]	3.20	541	0.001
Emotional Awareness	0.42	[0.28, 0.55]	6.16	541	<0.001
Trusting	<0.01	[−0.13, 0.14]	0.02	541	0.983
Not-Distracting	−0.09	[−0.17, −0.01]	−2.31	541	0.021

*N* = 548. Multiple linear regression with all six MAIA subscales entered simultaneously as predictors of the ELIA total score (mean of all 39 ELIA items). MAIA, Multidimensional Assessment of Interoceptive Awareness; ELIA, Emotion-Linked Interoceptive Awareness; β, standardized coefficients; CI, confidence interval.

The test–retest reliability of the ELIA scale was assessed in a subsample of 348 participants who passed all trap items and completed the scale at two time points. The ICC for the total score was 0.65 (95% CI: 0.59–0.71). Subscale-level ICCs were 0.64 for Positive Emotions (95% CI: 0.58–0.70), 0.60 for Anxiety (95% CI: 0.53–0.66), and 0.61 for Irritation (95% CI: 0.54–0.67).

Regarding practical usability, 65.9% of participants reported that the ELIA scale was easier to understand, and 66.8% found it easier to respond to than the MAIA. Both proportions were significantly greater than chance, as confirmed by the binomial tests (p < 0.001).

### 3.3 Associations between ELIA and established measures of interoception and psychological traits (Study 4)

Pearson correlations were computed between ELIA scores (total and three subscales) and established measures of interoception and psychological traits, including MAIA (total and six subscales), BPQ-BA, STAI-T, and TAS-20 (total and three subscales). Data from MBP non-practitioners who passed all trap items (*n* = 589; 248 women and 341 men; *M* = 49.23, SD = 12.17) were used. Table 3 reports the correlation coefficients and FDR-adjusted significance. 95% CIs for the correlation coefficients are provided in Supplementary Table 5.

**Table 3:** Bivariate correlations among ELIA, MAIA, BPQ-BA, STAI-T, and TAS-20.

	Et	Es1	Es2	Es3	Mt	Ms1	Ms2	Ms3	Ms4	Ms5	Ms6	B
Mt	**0.55**	**0.61**	**0.30**	**0.31**	—	—	—	—	—	—	—	—
Ms1	**0.46**	**0.55**	**0.20**	**0.21**	—	—	—	—	—	—	—	—
Ms2	**0.52**	**0.58**	**0.27**	**0.26**	—	—	—	—	—	—	—	—
Ms3	**0.55**	**0.53**	**0.40**	**0.41**	—	—	—	—	—	—	—	—
Ms4	**0.57**	**0.64**	**0.30**	**0.29**	—	—	—	—	—	—	—	—
Ms5	**0.46**	**0.57**	**0.15**	**0.19**	—	—	—	—	—	—	—	—
Ms6	**−0.40**	**−0.35**	**−0.35**	**−0.31**	—	—	—	—	—	—	—	—
B	**0.39**	**0.23**	**0.50**	**0.44**	**0.17**	**0.09**	**0.12**	**0.26**	**0.15**	0.07	**−0.28**	—
S	0.01	**−0.21**	**0.31**	**0.27**	**−0.29**	**−0.37**	**−0.30**	−0**.13**	**−0.27**	**−0.45**	−0.08	**0.32**
Tt	**0.10**	−0.06	**0.33**	**0.24**	**−0.13**	**−0.22**	**−0.11**	−0.02	**−0.10**	**−0.28**	**−0.16**	**0.32**
Ts1	**0.26**	0.09	**0.43**	**0.34**	0.02	−0.08	0.04	**0.10**	0.04	**−0.14**	**−0.21**	**0.37**
Ts2	0.07	−0.08	**0.27**	**0.22**	**−0.11**	**−0.19**	**−0.14**	<0.01	**−0.11**	**−0.24**	**−0.20**	**0.27**
Ts3	**−0.21**	**−0.24**	**−0.09**	**−0.13**	**−0.27**	**−0.28**	**−0.22**	**−0.23**	**−0.22**	**−0.29**	**0.12**	−0.02

*N* = 589. Cells report Pearson’s r. Two-tailed tests; significance was assessed using the Benjamini–Hochberg false discovery rate (FDR) correction across all pairwise correlations (bold indicates FDR-adjusted *p* < 0.05). Only the lower triangle is presented (diagonal omitted). Rows for ELIA variables (Et, Es1–Es3) are omitted because intercorrelations among ELIA scales were not a primary focus; for the same reason, within-MAIA correlations (i.e., between MAIA Total and its subscales, and among MAIA subscales) are also omitted. ELIA, Emotion-Linked Interoceptive Awareness; MAIA, Multidimensional Assessment of Interoceptive Awareness; BPQ-BA, Body Perception Questionnaire–Body Awareness; STAI-T, State–Trait Anxiety Inventory, Trait; TAS-20, 20-item Toronto Alexithymia Scale. Row and column labels utilize the following abbreviations: ELIA (Et, Total; Es1, Positive Emotions; Es2, Anxiety; Es3, Irritation); MAIA (Mt, Total; Ms1, Attention Regulation; Ms2, Body Listening; Ms3, Noticing; Ms4, Emotional Awareness; Ms5, Trusting; Ms6, Not-Distracting); B, BPQ-BA; S, STAI-T; TAS-20 (Tt, Total; Ts1, Difficulty Identifying Feelings; Ts2, Difficulty Describing Feelings; Ts3, Externally Oriented Thinking).

The ELIA total score and all subscales were positively correlated with the MAIA total score and all subscales except Subscale 6 (Not-Distracting), which was negatively correlated with all ELIA scores. The ELIA total and Subscale 1 (Positive Emotions) tended to show stronger correlations with MAIA, whereas Subscales 2 (Anxiety) and 3 (Irritation) showed weaker correlations.

All ELIA scores were positively correlated with BPQ-BA, with Subscales 2 and 3 exhibiting stronger correlations than the total score or Subscale 1.

Regarding STAI-T, MAIA scores (total and subscales) showed consistent negative correlations, whereas BPQ-BA showed positive correlations. Within the ELIA scale, Subscale 1 was negatively correlated with STAI-T, whereas Subscales 2 and 3 were positively correlated.

The MAIA total and subscale scores generally showed negative correlations with the TAS-20 total score and with the Subscale 2 (Difficulty Describing Feelings) and Subscale 3 (Externally Oriented Thinking) scores, although some exceptions were noted (e.g., MAIA Subscale 6 was positively correlated with TAS-20 Subscale 3). By contrast, the BPQ-BA showed positive correlations with the TAS-20 total score and with TAS-20 Subscales 1 and 2 scores.

The ELIA scores followed a distinct pattern: They were generally positively correlated with the TAS-20 total score and TAS-20 Subscales 1 and 2 scores, and negatively correlated with the TAS-20 Subscale 3 score. Notably, ELIA Subscales 2 and 3 scores showed relatively stronger positive correlations with the TAS-20 total score and TAS-20 Subscales 1 and 2 scores, whereas the ELIA total score and ELIA Subscale 1 score showed stronger negative associations with the TAS-20 Subscale 3 score.

### 3.4 Comparisons between MBP practitioners and matched controls (Study 4)

Group differences in ELIA, STAI, and TAS-20 scores between long-term MBP practitioners and non-practitioners were examined. A total of 589 non-practitioners and 168 practitioners who passed all trap items were included in the analysis. MBP practitioners reported sustained engagement in the following practices: yoga (62.5%), breathing meditation (8.3%), mindfulness (6.0%), zazen (6.0%), and other practices (17.2%). Notably, 51.2% had practiced for over 10 years, and 48.8% for over 5 years. A full breakdown of MBP types and practice durations is presented in Supplementary Table 4.

To minimize the potential confounding effects of age and gender, propensity score matching was conducted using 1:1 nearest-neighbor matching without replacement. Of the 589 eligible non-practitioners, 168 matched controls were selected. After matching, the two groups were well balanced in terms of age and gender, with a mean age of 50.36 years (SD = 11.62) for practitioners and 50.20 years (SD = 11.62) for controls and identical gender distributions (102 women in each group; 60.7%).

**Figure 3:**
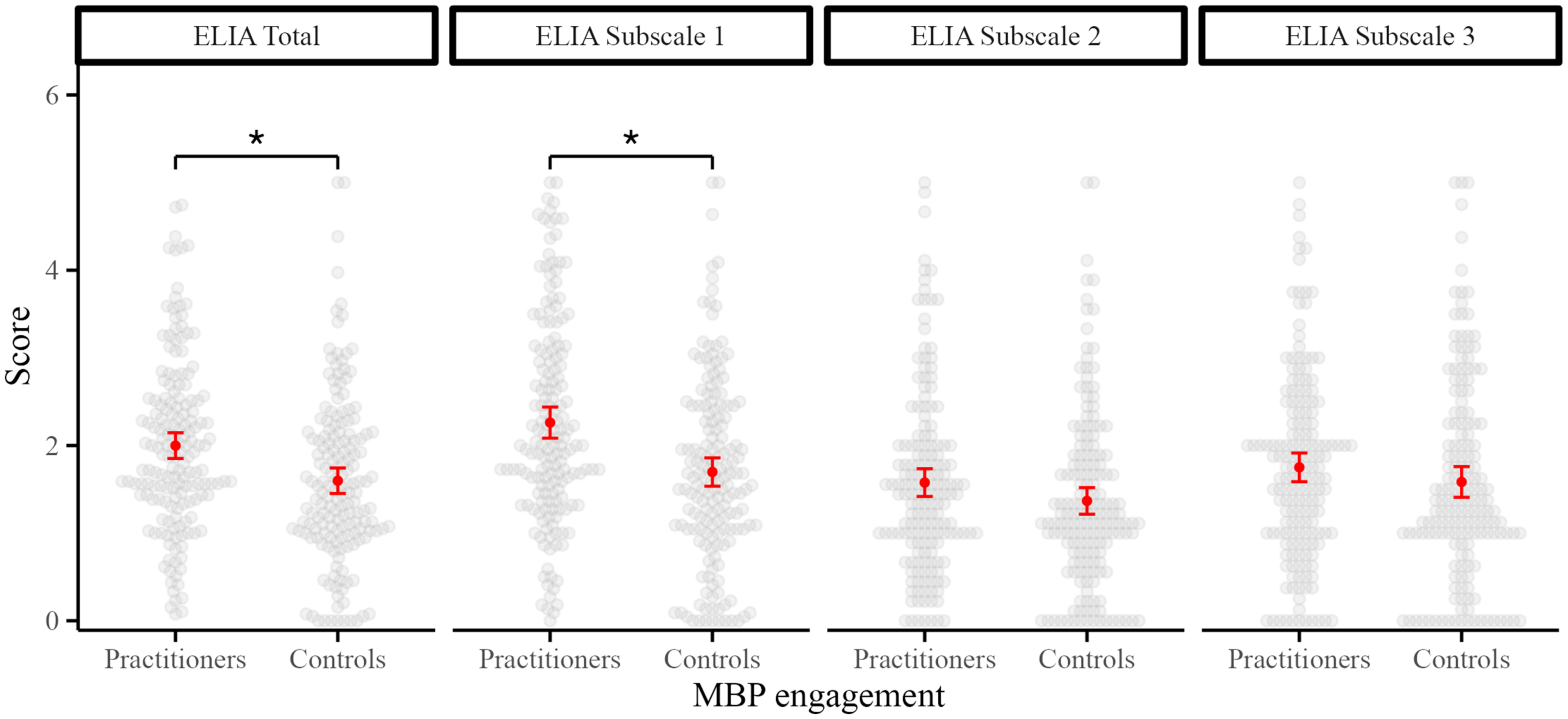
Group comparison of ELIA scores: MBP practitioners versus controls. ELIA scores in age- and gender-matched groups of MBP practitioners (≥5 years) and controls without MBP engagement (*N* = 336; *n* = 168 per group). Light gray dots represent individual participants, while red points indicate group means with 95% CI. Asterisks denote comparisons that were significant after Benjamini–Hochberg FDR correction of two-sample *t* tests (FDR-adjusted *p* < 0.05). ELIA total represents the average of all 39 items. Subscales 1, 2 and 3 index bodily awareness during positive emotions, anxiety, and irritation, respectively. ELIA, Emotion-Linked Interoceptive Awareness; MBP, mind–body practice; CI, confidence interval.

Group comparisons were conducted using independent samples t-tests in the matched samples (*n* = 168 per group). As shown in Figure 3, MBP practitioners scored significantly higher than matched controls on the ELIA total score [*t*(333.96) = 3.83, *p* < 0.001, d = 0.42] and Subscale 1 [Positive Emotions; (331.07) = 4.63, *p* < 0.001, d = 0.51]. Although practitioners also tended to score higher than controls on Subscales 2 (Anxiety) and 3 (Irritation), the differences were not statistically significant after FDR correction [Subscale 2: *t*(333.42) = 1.88, *p* = 0.081, d = 0.21; Subscale 3: *t*(332.41) = 1.37, *p* = 0.171, d = 0.1].

Trait anxiety and alexithymia were also compared between MBP practitioners and matched controls. Practitioners showed significantly lower trait anxiety than controls [STAI-T: *t*(333.93) = 2.51, *p* = 0.032, d = 0.27]. For alexithymia (TAS-20), only Subscale 3 (Externally Oriented Thinking) showed a significant group difference after FDR correction, with practitioners reporting lower scores than controls [*t*(330.24) = 3.09, *p* = 0.011, d = 0.34]. No significant differences were observed in the TAS-20 total score or the other subscales (ps > 0.10). All *p*-values reported reflect FDR-adjusted values.

### 3.5 Mediation analyses: ELIA scores as a mediator of MBP group differences (Study 4)

Mediation path models were fitted using SEM to examine whether ELIA scores mediated the relationship between long-term MBP engagement and psychological traits. Analyses were limited to psychological outcomes and ELIA indices that showed significant group differences after FDR correction (STAI-T, TAS-20 Subscale 3, ELIA total, and ELIA Subscale 1).

For STAI-T, when ELIA Subscale 1 was included as the mediator, the indirect effect was significant [95% BCa bootstrap CI (−2.62, −0.64)], whereas the direct effect was not [95% BCa bootstrap CI (−4.59, 0.67)]. By contrast, when the ELIA total score was included as the mediator, the indirect effect was not significant [95% BCa bootstrap CI (−0.77, 0.54)], whereas the direct effect remained significant [95% BCa bootstrap CI (−5.92, −0.58)].

For TAS-20 Subscale 3, indirect effects were significant when ELIA Subscale 1 was included as the mediator [95% BCa bootstrap CI (−0.90, −0.26)] and when the ELIA total score was included as the mediator [95% BCa bootstrap CI (−0.65, −0.13)]. The direct effect was not significant when ELIA Subscale 1 was included as the mediator [95% BCa bootstrap CI (−1.65, 0.03)] but was significant when the ELIA total score was included as the mediator [95% BCa bootstrap CI (−1.84, −0.17)].

**Figure 4:**
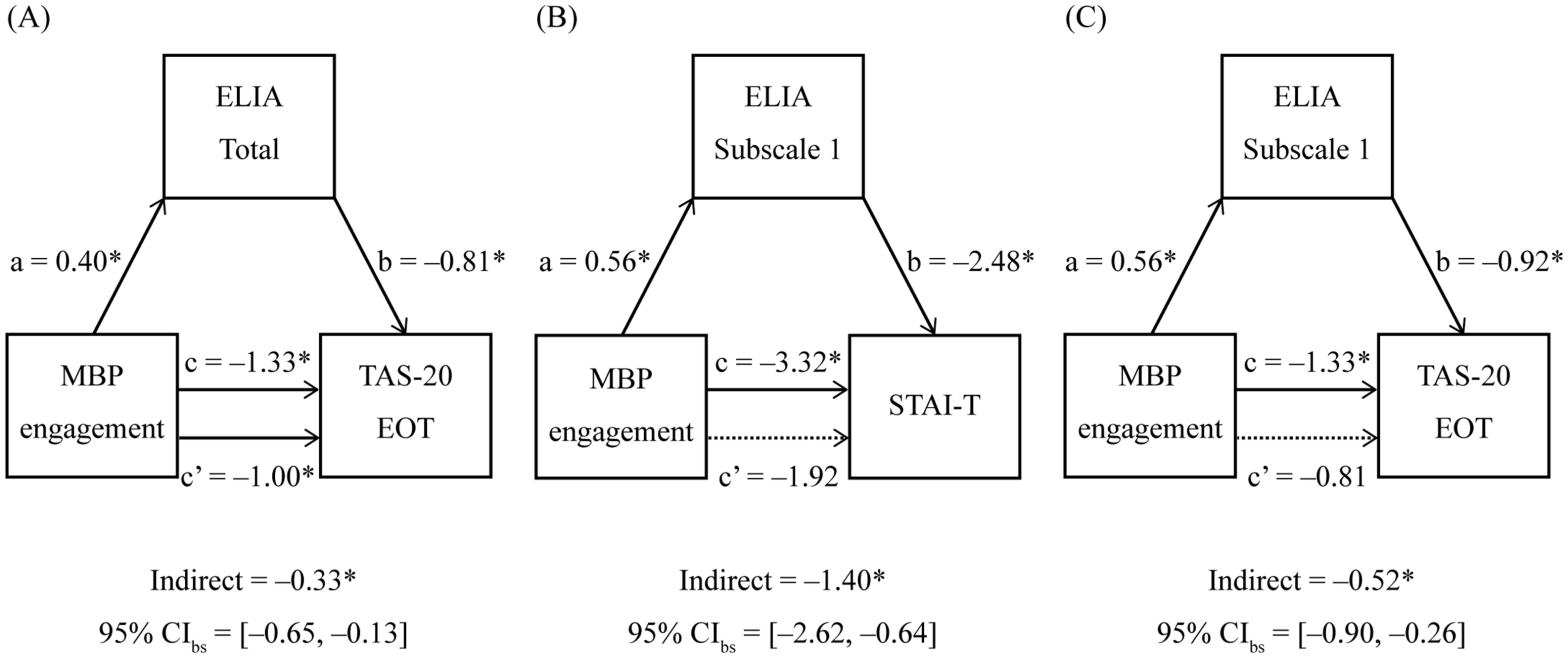
ELIA-mediated associations between MBP engagement and STAI-T/TAS-20 EOT. Panels **(A–C)** display mediator models assessing whether ELIA scores mediate the association of MBP engagement with psychological traits: **(A)** MBP engagement → ELIA Total → TAS-20 EOT; **(B)** MBP engagement → ELIA Subscale 1 → STAI-T; and **(C)** MBP engagement → ELIA Subscale 1 → TAS-20 EOT. Path notation: a (MBP engagement → ELIA), b (ELIA → trait), c′ (direct effect: MBP engagement → trait), and c (total effect: direct + indirect = c′ + a × b). Arrow labels indicate standardized path coefficients with 95% bias-corrected and accelerated (BCa) bootstrap CIs based on 5,000 resamples. The indirect effect (a × b) and its 95% BCa bootstrap CI are presented below each panel. Statistical significance for all displayed effects was determined by whether the 95% BCa bootstrap CI excludes zero; asterisks denote effects meeting this criterion. Solid lines indicate significant effects, while dashed lines indicate non-significant effects according to this CI-based criterion. *N* = 336. ELIA total represents the mean of 39 items; Subscale 1 indexes bodily awareness during positive emotions. MBP engagement was coded as follows: 0 = non-practitioner, 1 = practitioner. MBP, mind–body practice; ELIA, Emotion-Linked Interoceptive Awareness; STAI-T, State–Trait Anxiety Inventory (Trait); TAS-20, 20-Item Toronto Alexithymia Scale; EOT, Externally Oriented Thinking (TAS-20 subscale); CI, confidence interval.

Figure 4 presents the mediation models that yielded significant indirect effects. For completeness, Supplementary Figure 3 presents the mediation model with a non-significant indirect effect. It also reports the mediation effects for ELIA Subscales 2 and 3, for which no significant group differences were observed between long-term MBP practitioners and non-practitioners.

## 4 Discussion

This research aimed to advance our understanding of interoceptive sensibility by developing and validating a novel psychological scale, the ELIA, which uniquely focuses on bodily sensation awareness during distinct emotional experiences. Overall, these findings support the psychometric properties and theoretical relevance of the ELIA. Notably, the ELIA offers insights into how adaptive and maladaptive attentional forms of interoceptive sensibility can be conceptualized by differentiating bodily awareness across positive and negative emotional contexts. This distinction broadens discussions of interoceptive attentional style beyond mindfulness- and somatization-centered frameworks, such as those examined using the MAIA and BPQ-BA, by situating these styles within the dynamic interplay between interoception and emotion. Adopting this perspective may enhance our understanding of psychological well-being and guide the development of targeted interventions.

### 4.1 Factor structure and theoretical implications

The ELIA scale exhibited a bifactor structure, indicating that items across different emotional contexts load on a general factor, which is common to all items, and on a group-specific factor, which is shared by a subset of items. Exploratory bifactor analysis identified three specific subgroup factors corresponding to bodily sensations associated with positive emotions, anxiety, and irritation, alongside a general factor that spans these three subgroups. A follow-up confirmatory bifactor analysis in an independent sample further supported this structure, providing evidence for the theoretical coherence of the ELIA scale’s construct.

Although the subgroup factors exhibited relatively clear differentiation across emotional contexts, caution is warranted in interpreting the general factor as a cross-emotion interoceptive sensibility fully independent of specific emotional contexts. Indeed, the CFA loadings and ωHS values indicated that the Positive Emotions subfactor contributed minimal unique variance beyond the general factor. This pattern may be attributable to two factors: Positive-emotion items constitute a majority of the scale (22 of 39), and positive emotional experiences are reported more frequently than negative ones in daily life (Oishi et al., 2007; Scollon et al., 2004). Alternatively, it may suggest that awareness of bodily sensations during positive emotional states centrally contributes to shaping general interoceptive sensibility, particularly in non-clinical populations. This interpretation aligns with evidence that the MAIA, which in the present work showed a relatively stronger positive correlation with the ELIA Positive Emotions subscale, is most centrally positioned within a global interoceptive sensibility construct captured by widely used self-report measures (Todd et al., 2022). Accordingly, unless further validation studies using this scale or its derivatives provide contrary evidence, this general factor should be referred to as “positivity-weighted cross-emotion interoceptive sensibility.”

Beyond the general factor, the subgroup factors raise questions regarding their correspondence with established emotion models. Although ELIA items were designed to span a broad spectrum of emotional experiences consistent with the circumplex model’s four-quadrant structure (Russell, 1980), the resulting factor structure did not support the four distinct categories predicted by the model. Items related to positive emotions (e.g., happiness, excitement, relaxation, and contentment) were loaded onto a single subfactor, whereas negative high-arousal states (e.g., anxiety and irritation) formed two distinct subfactors. By contrast, items related to sadness and boredom exhibited inconsistent and heterogeneous loading patterns, rendering them difficult to organize into a coherent substructure. One possible explanation is that negative low-arousal states (e.g., sadness) are accompanied by attenuated or downregulated bodily sensations, reducing the salience and consistency of interoceptive cues available for self-report and, in turn, yielding weaker and more heterogeneous item loadings. This account aligns with neuroimaging evidence demonstrating relative deactivation of the right insula, a region implicated in interoceptive processing, when viewing sadness-inducing films (Farb et al., 2010). Additional investigation is needed to assess whether attenuated interoceptive signals during low-arousal negative states are reflected in self-report, and to clarify why positive emotions with distinct arousal levels, typically differentiated in the circumplex model (e.g., excitement versus relaxation), loaded onto a single subfactor in the present data. These findings do not undermine the circumplex model’s utility but suggest that bodily awareness associated with emotional experiences may not map neatly onto a two-dimensional valence-arousal space.

Items related to anxiety and irritation formed distinct subfactors. Both emotions are typically positioned in the circumplex model’s negative, high-arousal quadrant (Russell, 1980), and such negatively valenced high-arousal states may be experienced as particularly arousing. Heightened perceived arousal may increase the salience of bodily sensations, making bodily awareness related to these emotions more likely to stand out. Consistent with this account, image-based studies have reported an asymmetrical valence bias in arousal judgments, with negative high-arousal stimuli rated as more arousing than positive stimuli (Grühn and Scheibe, 2008). Neuroimaging work has further demonstrated that viewing films that elicit high perceived arousal is associated with stronger insula activation, a key region for interoceptive processing (Wang et al., 2023). Collectively, these findings suggest that elevated perceived arousal may have contributed to the distinct subfactors observed for anxiety and irritation. A separate question is why anxiety and irritation split into two subfactors rather than clustering together. One possibility is that these emotions differ qualitatively in bodily-sensation profiles, yielding separable item clusters. In Study 1, co-occurrence networks suggested partially distinct patterns between the two emotions: Anxiety included dryness in the mouth or throat and tightening in the chest, whereas irritation included hot sensations in the head and gastric discomfort or nausea. Alternative explanations should also be considered. The separation may be attributable to anxiety- and irritation-specific physiological responses, though emotion-specific physiological signatures remain debated (Barrett, 2017; Hoemann et al., 2020). Accordingly, whether the observed separation primarily reflects perceived emotional arousal, physiological responses, or qualitative sensation profiles remains an open question.

Finally, in light of the growing criticism that existing measures such as the MAIA and BPQ-BA struggle to capture a unified construct of interoception (Desmedt et al., 2022; Vig et al., 2022), and that even their subscales may not converge on a single dimension (Desmedt et al., 2022; Ferentzi et al., 2021), the ELIA offers a more coherent account of bodily awareness in emotional contexts. Compared to the MAIA’s Emotional Awareness subscale, which comprises only five relatively abstract items, the ELIA provides finer granularity by grouping concrete and emotion-specific bodily sensations into three distinct emotional contexts. Thus, the bifactor structure supports the idea that bodily awareness in emotional contexts constitutes a unified yet differentiated domain, thereby offering meaningful contributions to the theoretical models of interoceptive sensibility.

### 4.2 Relationships with existing interoceptive scales: adaptive and maladaptive attention

Correlational analyses revealed distinct association patterns between ELIA, particularly its subscales, and established measures of interoception and related traits. The ELIA total and subscale scores showed the strongest associations with the MAIA Emotional Awareness subscale, supporting ELIA’s content validity as a measure of emotion-linked bodily awareness. Moderate positive correlations were also observed with the MAIA Noticing subscale, consistent with ELIA’s emphasis on the capacity to perceive bodily sensations. Although the MAIA Not-Distracting subscale reached statistical significance, its negligible effect size (*β* < 0.10) suggests minimal conceptual overlap with ELIA. Additionally, weak-to-moderate positive correlations were typically observed among the ELIA, MAIA, and BPQ-BA scales, consistent with previous findings that these instruments capture partially overlapping yet distinct facets of interoceptive sensibility (Desmedt et al., 2022; Vig et al., 2022).

Among the ELIA subscales, Subscale 1 (reflecting bodily awareness during positive emotional states) showed the strongest and most consistent positive correlations with MAIA (excluding Not-Distracting). Additionally, the MAIA total score and most subscales were negatively associated with trait anxiety (STAI-T) and alexithymia (TAS-20 total and Subscales 2 and 3), reinforcing its role as an indicator of adaptive interoceptive attention (Mehling, 2016; Trevisan et al., 2021, 2023). Building on this pattern, the ELIA findings suggest that heightened bodily awareness during positive emotions may reflect a more adaptive facet of interoceptive sensibility. Conversely, the MAIA Not-Distracting subscale, which is psychometrically distinct from other MAIA dimensions (Desmedt et al., 2022; Ferentzi et al., 2021), showed inverse or inconsistent associations with the trait measures and ELIA scores. This pattern suggests that the Not-Distracting subscale may not align well with adaptive interoceptive constructs, at least in non-clinical Japanese populations.

By contrast, ELIA Subscales 2 and 3 (reflecting bodily awareness during anxiety and irritation, respectively) were more strongly associated with BPQ-BA, a measure generally discussed in relation to somatic hypervigilance and somatosensory amplification (Cabrera et al., 2018). These subscales also showed positive associations with trait anxiety and alexithymia, particularly TAS-20 Subscales 1 and 2. Collectively, these results suggest that heightened bodily awareness during negative emotional states may represent a more maladaptive facet of interoceptive sensibility. Overall, these correlational profiles indicate the ELIA’s potential to distinguish between adaptive and maladaptive interoceptive attentional styles as a function of emotional valence. They also suggest that the attentional-style framework may be applicable within a single sub-dimension of interoceptive sensibility, rather than solely by contrasting distinct interoceptive scales.

When considering the relationship between bodily awareness across emotional contexts and attentional styles, particularly regarding adaptiveness, several caveats are warranted. The ELIA assesses the extent to which individuals notice bodily sensations during specific emotions, whereas recent theoretical work has emphasized that adaptiveness depends on both the quality of interoceptive attention and the nature of appraisal of interoceptive information (Joshi et al., 2021). Thus, the degree of bodily awareness should not be conflated with the deployment or regulation of interoceptive attention, nor used as a direct proxy for adaptiveness. From this perspective, the positive associations of ELIA Subscales 2 and 3 (Anxiety and Irritation) with trait anxiety and alexithymia should be interpreted cautiously, because these subscales do not directly assess attentional quality or interpretative processes. Additionally, these correlations may partially reflect a higher frequency or greater intensity, or both, of negative emotional episodes among individuals with higher trait anxiety or higher alexithymia, which were not measured or controlled in the present work. Therefore, the correlational data alone cannot determine whether the observed associations reflect more frequent/intense negative emotional episodes, heightened bodily awareness, hypervigilant monitoring, or catastrophic interpretations. In sum, adaptiveness may be better conceptualized as emerging from the joint contribution of attentional and interpretative processes, which may correlate with bodily awareness at the trait level while remaining conceptually distinct from it.

### 4.3 Mind–body practice, interoception, and psychological health

Group comparisons between MBP practitioners and non-practitioners, together with mediation analyses involving ELIA scores, further underscored the applied value of bodily awareness across positive and negative emotional contexts. Matched-sample comparisons revealed that long-term MBP practitioners reported higher ELIA scores and lower levels of trait anxiety and alexithymia than non-practitioners. Although practitioners scored higher on all ELIA subscales, including those related to anxiety and irritation, the largest and statistically significant differences were observed for bodily awareness during positive emotional states. This pattern suggests that engaging in MBPs may be associated with greater bodily awareness during positive emotions, consistent with prior work indicating that MBPs enhance interoceptive abilities by fostering adaptive bodily awareness (Bornemann et al., 2015; Mehling et al., 2018). Nevertheless, these group differences should be interpreted cautiously, as they may partially reflect unmeasured covariates (e.g., attentional quality and interpretive style) that differ between practitioners and non-practitioners and could confound the observed ELIA differences.

Moreover, mediation analyses revealed that greater bodily awareness during positive emotional states mediated the association between MBP engagement and lower levels of both trait anxiety and externally oriented thinking. Importantly, these mediation effects appeared to be valence-selective. They emerged for the ELIA total score and the Positive Emotions subscale score, but not for the negative-emotion subscale scores (Anxiety and Irritation). This pattern supports the interpretation that the general factor captures cross-emotion interoceptive sensibility, with positive emotional contexts contributing more robustly. Accordingly, the Positive Emotions subscale may be better conceptualized as capturing the core of the general factor rather than representing a distinct, independent dimension. Heightened bodily awareness during positive emotional states may therefore be particularly important for interpreting the observed associations between MBP engagement and psychological well-being.

Additionally, this interpretation aligns with the Mindfulness-to-Meaning Theory, particularly its account of savoring (Garland et al., 2015). The theory posits that mindfulness practice cultivates psychological space for positive reappraisal. The resulting positive framing, in turn, orients attention toward positive aspects of daily life, facilitating savoring and amplifying positive emotions. Over time, these reciprocal associations between positive appraisals and positive emotions are theorized to produce an iterative upward spiral. Although the theory identifies interoceptive attention as a key process whereby mindfulness shapes appraisal, it does not explicitly address bodily awareness within positive emotional contexts. Collectively, the present findings may extend the theory by elaborating savoring in terms of emotion-linked bodily awareness, potentially informing more efficient emotion regulation strategies. As mindfulness-to-meaning–based interventions have been applied to varied clinical problems, including addictive behavior, chronic pain, and psychiatric symptoms (Parisi et al., 2022), this refinement may be particularly relevant for future clinical translation.

### 4.4 Limitations and future directions

Despite these contributions, the present research has several limitations. First, items related to low-arousal negative emotions (e.g., sadness and boredom) were excluded during scale development. This decision was intended to achieve a simple subgroup-factor structure while preserving the overarching general factor. However, the resulting scale does not capture the interoceptive awareness associated with these emotional states. Restricting the range of emotional contexts represented in the item pool may narrow construct coverage. Consequently, the scale may place disproportionate emphasis on bodily awareness within a limited subset of emotional contexts, indicating construct underrepresentation owing to item-selection decisions. Future research should explore alternative analytical strategies or item formats to better assess bodily awareness across a broader range of emotional contexts, particularly those involving negative and low-arousal states.

Second, although the ELIA scale targets awareness of bodily sensations, whether respondents genuinely perceive these sensations or merely report their beliefs or assumptions regarding their bodily responses remains unclear. Thus, ELIA scores may not be strongly correlated with behavioral or physiological indices of interoception (Garfinkel et al., 2015).

Third, the test–retest reliability of the ELIA scale was moderate (ICC ≈ 0.60), which is within the acceptable range for self-report psychological measures but not especially high. One plausible explanation is that the detectability of bodily sensations may fluctuate within individuals depending on the social context or situational frequency of specific emotions, particularly those that are less frequently experienced in daily life.

Fourth, the sample exclusively comprised non-pregnant Japanese adults without neurological, autonomic, or psychiatric disorders or disabilities, limiting the findings’ generalizability. Studies in younger populations, pregnant individuals, and individuals with disabilities are needed to evaluate the scale’s applicability beyond the present research’s eligibility criteria. Additionally, because participants were recruited from an opt-in online panel (i.e., a non-probability sample), the sample may be subject to selection and coverage biases and may not adequately represent the target Japanese adult population (Bethlehem, 2010; Freese and Jin, 2025).

Fifth, cultural and clinical generalizability warrants careful examination. Prior cross-cultural research has documented cultural differences in the frequency of pleasant emotional experiences, with European American respondents reporting such experiences more frequently than Japanese respondents (Oishi et al., 2007; Scollon et al., 2004). Further, cross-cultural differences have been reported in the factor structure of self-report measures of interoceptive sensibility, including the MAIA’s language- and culture-specific factorial structures (Mehling et al., 2012; Shoji et al., 2018; Todd et al., 2020). Therefore, cross-national replications of ELIA are necessary to assess its cultural robustness. Additional validation in clinical populations (such as individuals with anxiety or depressive disorders) is required to evaluate the scale’s clinical sensitivity and relevance.

Finally, the cross-sectional nature of this research precludes causal inferences (for example, whether long-term engagement in MBP leads to changes in emotionally linked interoceptive awareness). Future longitudinal and intervention studies are essential for clarifying the temporal and causal dynamics linking interoception, emotional experience, and psychological health. Addressing these limitations in future research will not only enhance the generalizability of the ELIA scale but also extend its utility across diverse populations and applied contexts.

## 5 Conclusion

In summary, the present research introduced and validated the ELIA scale, a novel measure that focuses on bodily awareness in emotional contexts. Although the scale encompasses a range of emotional states, it demonstrates a bifactor structure; this suggests that all items tap into a single underlying construct of bodily awareness specifically linked to emotional experiences. Importantly, the results obtained using the ELIA scale suggest that stronger awareness of bodily sensations during positive emotions may reflect adaptive aspects of interoceptive sensibility; meanwhile, stronger awareness during negative emotions may be related to maladaptive aspects. Its ability to distinguish between emotional valences in bodily awareness provides a useful framework for examining individual differences in psychological functioning. Moreover, the ELIA scale consists of concrete emotion-specific items derived from free-text descriptions provided by community samples, which are rated as easy to understand and respond to, highlighting its practical advantages for clinical, intervention, and research applications. Thus, the ELIA offers promising insights for advancing theoretical models of interoception and providing interventions aimed at enhancing emotional regulation and psychological health. To enhance its translational relevance to practice, future research should evaluate the ELIA’s clinical validity within diagnostically characterized clinical samples. In parallel, cross-cultural validation is warranted to determine whether the ELIA’s factor structure and clinical correlates replicate across languages and cultural contexts.

## Data Availability

The datasets presented in this study can be found in online repositories. The names of the repository/repositories and accession number(s) can be found at: https://doi.org/10.6084/m9.figshare.30094345.
