# Supplementary material for: Understanding interoception across emotional contexts: development and validation of the Emotion-Linked Interoceptive Awareness Scale
Source: Front Psychol. 2026 Mar 18;17:1757948. doi: 10.3389/fpsyg.2026.1757948 (PMC13040479; doi:10.3389/fpsyg.2026.1757948)
Supplement: Supplementary file 1 [file Table_1.docx]

**Supplementary Materials**

1. **Item Development Based on Free-Text Data (Study 1)**

The Emotion-Linked Interoceptive Awareness (ELIA) scale was designed to assess individuals’ tendencies to notice their own bodily sensations, particularly in specific emotional contexts, as described in the main manuscript. Four studies were conducted to develop and validate ELIA. The principal aim of this work was to create an interoceptive scale that focuses on bodily sensations under specific emotional contexts; additionally, this research sought to ensure that the scale is readily understandable and answerable by the general public with no medical conditions or specialized training.

To achieve these aims, the scale was not constructed based on any particular theory or views of expert mind-body practitioners; instead, the item content was designed to reflect laypersons’ everyday bodily sensations as faithfully as possible. Free-text descriptions were obtained from 2,500 Japanese adults (1,250 women and 1,250 men) describing the bodily sensations they typically experienced when each of the following eight emotions was elicited: happiness, excitement, relaxation, contentment, anxiety, irritation, sadness, and boredom. Using co-occurrence network analysis with community detection, the words and combinations of words most frequently employed to describe emotion-linked bodily sensations were identified, and, on that basis, 66 candidate items were generated. In the main manuscript, the details of the methods and results of Study 1 were omitted due to space constraints and word limits, and to maintain the continuity of presentation. This section discusses the omitted details.

**Method**

Analyses were conducted separately for each emotion-specific sub-corpus. The analytic pipeline proceeded as follows: (i) sentence tokenization; (ii) token normalization; (iii) part-of-speech (POS) filtering to retain only nouns, verbs, adjectives, and adverbs; (iv) the removal of tokens listed in a predefined stopword list; (v) the selection of the 50 most frequent tokens in the sub-corpus; and (vi) the construction of a word co-occurrence network and community detection. All analyses were performed using Python (v3.12.2).

**Tokenization/Normalization and Feature Selection**

Sentence tokenization and POS tagging were performed with spaCy (Honnibal et al., 2023) using the GiNZA Japanese pipeline (Megagon Labs, 2024). After tokenization, the tokens were normalized with neologdn (Ikegami, 2025) to prevent the inflation of token counts due to orthographic variation. The tokens were filtered to retain only nouns, verbs, adjectives, and adverbs, excluding particles, conjunctions, and other functional words. Additionally, the following 41 terms were removed as stopwords, based on an a priori assumption that they are either very frequent, semantically generic lexical items (e.g., verbs corresponding to English “see,” “say,” “get,” “feel,” “think,” “consider”), or directly denote the target emotions and would therefore offer limited discriminative value for constructing emotion-specific co-occurrence networks: 成る, 為る, 有る, 居る, 言う, 見る, 見える, 思う, 考える, 分かる, 行く, 来る, 仕舞う, 感じる, 感ずる, 事, 物, 時, 際, 様, 方, 為, 等, 感じ, 感, 感覚, 状態, 感情, 気持ち, 気分, 気, こと, よう, 嬉しい, わくわく, リラックス, 満ち足りる, 悲しい, うんざり, 不安, 苛々. The 50 most frequent tokens were selected within each emotion-specific sub-corpus from the tokens remaining after POS filtering and stopword removal.

**Co-occurrence Network Analysis and Community Detection**

For each emotion, the pairwise co-occurrence between the top 50 tokens was computed at the response level (per participant, per emotion). Two tokens were defined as co-occurring if both appeared at least once in the same participant’s free-text response; for each pair, the Jaccard coefficient was calculated over the set of responses in that emotion-specific sub-corpus. Edges were included when J > .10, yielding an undirected, weighted graph with edge weights equal to J; tokens with no above-threshold co-occurrences were excluded. Communities were identified using the Clauset–Newman–Moore greedy modularity maximization (Clauset et al., 2004), as implemented in NetworkX’s greedy_modularity_communities (Hagberg et al., 2008). All function parameters were set to default settings.

**Results**

Supplementary Figure 1 (panels A–H) depicts the co-occurrence networks of the eight emotions and the communities identified within them. The nodes (circles) represent words, and edges represent co-occurrence links; thicker edges indicate higher Jaccard coefficients. Distinct communities are shown in different colors. To enhance readability, community-level English descriptions are added to the figure as brief glosses summarizing each community’s theme; they do not constitute translations of individual Japanese words. Notably, the analyzed text data and item pools were in Japanese. Guided by the co-occurrence relations between words within each community, one (or, in some cases, several) candidate item(s) were drafted per community. Throughout item development, all items adhered to a uniform format: “When I feel [emotion], I notice [bodily sensations].” Expressions clearly unrelated to bodily sensations or judged ambiguous (for example, “my mind settles down,” “my facial expression brightens,” “I feel motivated”) were excluded. This process yielded 66 items.


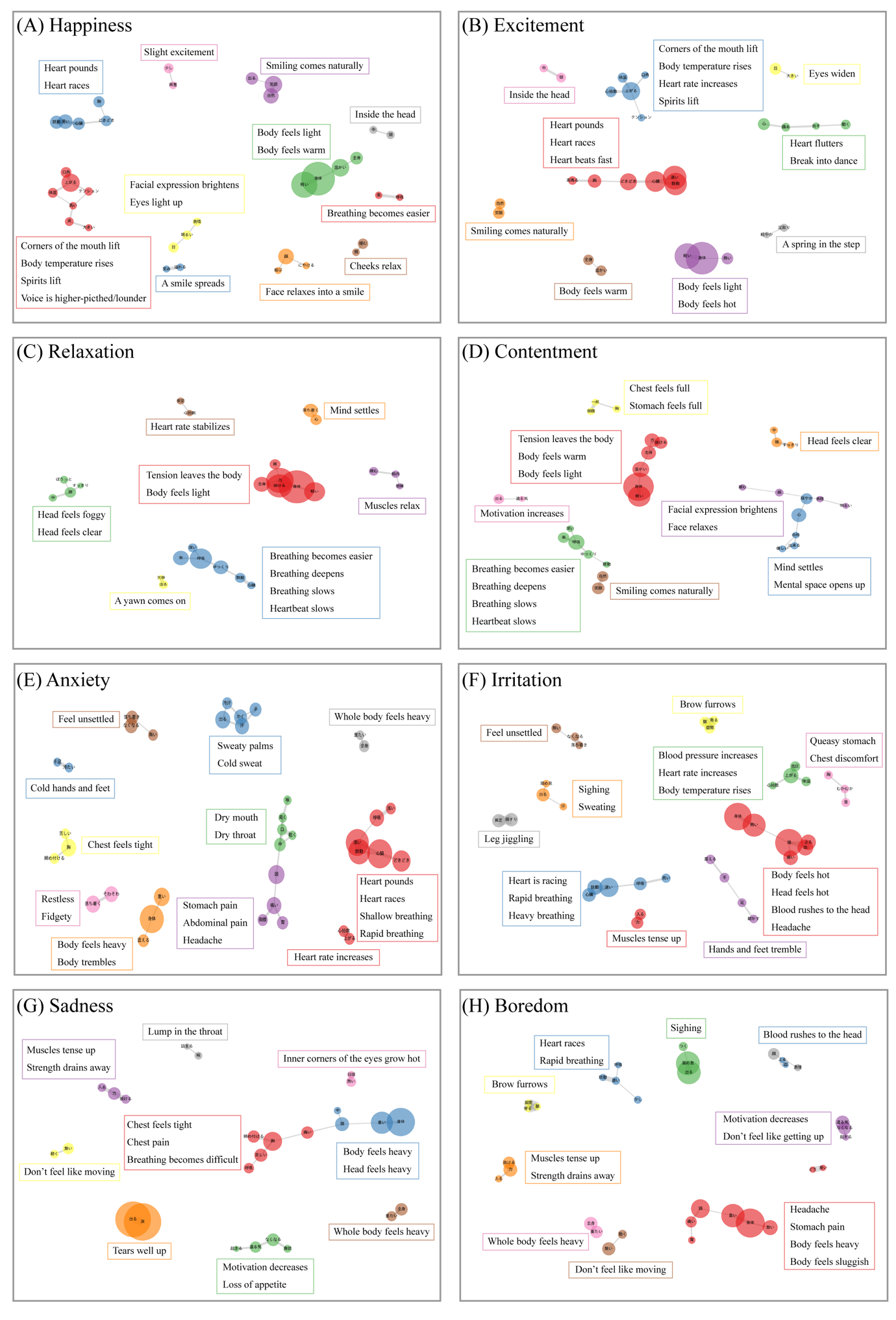


**Supplementary Figure 1**. Frequent word co-occurrence networks of bodily sensations across eight emotions.

Co-occurrence networks of the 50 most frequent words utilized to describe bodily sensations accompanying eight emotions (Panels A–H). Nodes represent words, while larger nodes indicate higher word frequency in the corpus. Edges indicate co-occurrence weighted by the Jaccard coefficient, while only edges with J > .10 are displayed (pairs with J ≤ .10 were pruned). Thicker edges indicate higher Jaccard coefficients. Distinct communities, presented in different colors, were detected using the Clauset–Newman–Moore greedy modularity maximization. Community-level English glosses summarize themes and do not constitute literal translations of individual Japanese words.

1. **ELIA Items and Factor Analysis Results (Study 2)**

Using the 66 candidate items generated in Study 1, Study 2 collected responses from 1,000 Japanese adults (500 women and 500 men). After excluding inattentive respondents identified with a trap item, data from 831 participants were randomly split into two independent subsamples, one (n = 416) for exploratory factor analysis (EFA) and the other (n = 415) for confirmatory factor analysis (CFA). The EFA employed a bifactor modeling approach with the Direct Schmid–Leiman (DSL) transformation, which yielded a structure comprising one general factor and three subfactors across 39 items. Replicability of this bifactor structure was then evaluated in an independent subsample via CFA, which supported the model. Due to space constraints and word limits, the main manuscript does not include the full item set (including items excluded during factor analysis) and EFA/CFA loading matrices, which are discussed in this section.

Supplementary Table 1 presents the EFA and CFA results of the ELIA items. The table reports each item’s factor loadings, the latent factor correlations in the CFA model, and the items removed during the EFA. For readability, items belonging to the same emotion are grouped together, and the bodily sensation phrases for each emotion are listed in separate cells. Notably, each item was presented in the following form: “When I feel [emotion], I notice [bodily sensations].”

| **Supplementary Table 1.** EFA/CFA factor loadings of the ELIA | | | | | | | | | | | |
| --- | --- | --- | --- | --- | --- | --- | --- | --- | --- | --- | --- |
|  | Factor loadings | | | | | | | | | | |
| Items | General  factor | |  | Subgroup factor 1 | |  | Subgroup factor 2 | |  | Subgroup factor 3 | |
|  | EFA | CFA |  | EFA | CFA |  | EFA | CFA |  | EFA | CFA |
| **When I feel happy, I notice** | | | | | | | | | | | |
| my body temperature rising and warmth spreading through my whole body. | .57 | .80* |  | .51 | –.06 |  | .02 | — |  | .05 | — |
| my heart rate increasing and my heart pounding in my chest. | .60 | .73* |  | .48 | –.05 |  | .08 | — |  | .04 | — |
| my body feeling light. | .60 | .84* |  | .55 | .03 |  | –.06 | — |  | .11 | — |
| my breathing becoming easier. | .61 | .81* |  | .52 | –.09 |  | –.03 | — |  | .12 | — |
| **When I feel excited, I notice** | | | | | | | | | | | |
| my body temperature rising and warmth spreading through my whole body. | .60 | .80* |  | .55 | –.07 |  | .08 | — |  | –.02 | — |
| my body feeling light. | .57 | .76* |  | .55 | .11 |  | –.05 | — |  | .07 | — |
| my eyes opening wide. | .59 | .74* |  | .48 | –.08 |  | .11 | — |  | –.01 | — |
| a spring in my step. | .56 | .76* |  | .51 | .13 |  | –.01 | — |  | .06 | — |
| **When I feel relaxed, I notice** | | | | | | | | | | | |
| my body feeling light. | .55 | .80* |  | .58 | .29* |  | –.06 | — |  | .03 | — |
| my breathing becoming slower, deeper, and easier. | .55 | .79* |  | .56 | .38* |  | –.07 | — |  | .06 | — |
| my heartbeat slowing and my heart rate becoming steady. | .57 | .79* |  | .51 | .31* |  | –.03 | — |  | .09 | — |
| my head feeling clear. | .54 | .75* |  | .55 | .30* |  | –.11 | — |  | .10 | — |
| my muscles releasing tension. | .51 | .75* |  | .50 | .31* |  | –.05 | — |  | .06 | — |
| **When I feel content, I notice** | | | | | | | | | | | |
| the tension leaving my whole body. | .60 | .80* |  | .57 | –.20 |  | .07 | — |  | –.04 | — |
| my body feeling light. | .60 | .85* |  | .64 | –.15 |  | –.01 | — |  | –.02 | — |
| warmth spreading through my whole body. | .60 | .83* |  | .61 | –.28* |  | .11 | — |  | –.12 | — |
| my breathing becoming slower, deeper, and easier. | .60 | .88* |  | .63 | –.08 |  | .04 | — |  | –.08 | — |
| my heartbeat slowing. | .62 | .85* |  | .60 | –.19 |  | .09 | — |  | –.07 | — |
| my face relaxing into a natural smile. | .50 | .79* |  | .56 | –.04 |  | <.01 | — |  | –.05 | — |
| my head feeling clear. | .61 | .83* |  | .63 | –.15 |  | .03 | — |  | –.04 | — |
| my chest feeling full. | .58 | .84* |  | .56 | –.15 |  | .11 | — |  | –.09 | — |
| a comfortable fullness in my stomach. | .53 | .70* |  | .48 | –.20 |  | .12 | — |  | –.07 | — |
| **When I feel anxious, I notice** | | | | | | | | | | | |
| my breathing becoming shallow. | .68 | .49* |  | .07 | — |  | .48 | .67* |  | .13 | — |
| my palms getting sweaty. | .60 | .38* |  | .02 | — |  | .50 | .68* |  | .08 | — |
| a cold sweat breaking out. | .66 | .43* |  | .02 | — |  | .49 | .70* |  | .14 | — |
| my mouth and throat becoming dry. | .63 | .41* |  | .04 | — |  | .50 | .68* |  | .08 | — |
| a headache. | .65 | .43* |  | .03 | — |  | .48 | .71* |  | .13 | — |
| stomach pain. | .58 | .37* |  | .04 | — |  | .49 | .66* |  | .05 | — |
| my body trembling. | .66 | .39* |  | .06 | — |  | .54 | .72* |  | .06 | — |
| a tight, constricted feeling in my chest. | .66 | .40* |  | .03 | — |  | .48 | .69* |  | .15 | — |
| my hands and feet getting cold. | .60 | .43* |  | .06 | — |  | .55 | .69* |  | –.01 | — |
| **When I feel irritated, I notice** | | | | | | | | | | | |
| a rush of blood to my head. | .62 | .40* |  | .01 | — |  | .09 | — |  | .52 | .74* |
| my body temperature rising and my whole body feeling hot. | .67 | .45* |  | <.01 | — |  | .14 | — |  | .53 | .71* |
| my head feeling hot. | .67 | .43* |  | .05 | — |  | .12 | — |  | .50 | .72* |
| my heart beating faster. | .68 | .46* |  | <.01 | — |  | .12 | — |  | .56 | .74* |
| my breathing speeding up. | .67 | .47* |  | .07 | — |  | .06 | — |  | .54 | .72* |
| my breathing becoming heavy. | .67 | .41* |  | .02 | — |  | .06 | — |  | .58 | .73* |
| sweat breaking out. | .61 | .32* |  | –.02 | — |  | .14 | — |  | .49 | .73* |
| my muscles tensing up. | .64 | .48* |  | .04 | — |  | .08 | — |  | .52 | .69* |
|  |  |  |  |  |  |  |  |  |  |  |  |
| Latent factor correlations in the CFA model | | | | | | | | | | | |
|  | General  factor | |  | Subgroup factor 1 | |  | Subgroup factor 2 | |  | Subgroup factor 3 | |
| General factor | — | |  |  | |  |  | |  |  | |
| Subgroup factor 1 | .00 | |  | — | |  |  | |  |  | |
| Subgroup factor 2 | .00 | |  | .00 | |  | — | |  |  | |
| Subgroup factor 3 | .00 | |  | .00 | |  | .63* | |  | — | |
|  |  |  |  |  |  |  |  |  |  |  |  |
| Removed items during EFA | | | | | | | | | | | |
| **When I feel happy, I notice** | | | | | | | | | | | |
| the corners of my mouth lifting and my cheeks relaxing into a natural smile. | | | | | | | | | | | |
| **When I feel excited, I notice** | | | | | | | | | | | |
| my heart racing and pounding in my chest. | | | | | | | | | | | |
| the corners of my mouth lifting into a natural smile. | | | | | | | | | | | |
| **When I feel relaxed, I notice** | | | | | | | | | | | |
| the tension leaving my whole body. | | | | | | | | | | | |
| my head feeling foggy. | | | | | | | | | | | |
| **When I feel anxious, I notice** | | | | | | | | | | | |
| my heart racing and pounding in my chest. | | | | | | | | | | | |
| my whole body feeling heavy. | | | | | | | | | | | |
| **When I feel irritated, I notice** | | | | | | | | | | | |
| my hands trembling. | | | | | | | | | | | |
| a sigh escaping me. | | | | | | | | | | | |
| my brow furrowing. | | | | | | | | | | | |
| **When I feel sad, I notice** | | | | | | | | | | | |
| a tight, painful feeling in my chest. | | | | | | | | | | | |
| my whole body feeling heavy. | | | | | | | | | | | |
| my breathing becoming difficult. | | | | | | | | | | | |
| my head feeling heavy. | | | | | | | | | | | |
| the strength draining from my body. | | | | | | | | | | | |
| tears welling up in my eyes. | | | | | | | | | | | |
| the inner corners of my eyes growing hot. | | | | | | | | | | | |
| a lump in my throat. | | | | | | | | | | | |
| **When I feel bored, I notice** | | | | | | | | | | | |
| my whole body feeling heavy. | | | | | | | | | | | |
| my head feeling heavy. | | | | | | | | | | | |
| my body feeling sluggish. | | | | | | | | | | | |
| my heart beating faster. | | | | | | | | | | | |
| my breathing speeding up. | | | | | | | | | | | |
| a sigh escaping me. | | | | | | | | | | | |
| the strength draining from my body. | | | | | | | | | | | |
| my brow furrowing. | | | | | | | | | | | |
| a rush of blood to my head. | | | | | | | | | | | |
| Note. Respondent instructions: The following items describe bodily sensations that accompany specific emotions. For each emotion, indicate how often you notice the bodily sensations when you experience that emotion by selecting a number from 0 (Never) to 5 (Always). EFA: N = 416; CFA: N = 415. The EFA employed a bifactor exploratory approach using the Direct Schmid–Leiman transformation. For the CFA, a bifactor model comprising one general factor and three subgroup factors was estimated using robust maximum likelihood. Item-to-factor assignments followed the EFA solution: Each item loaded on the general factor and on one subgroup factor, with cross-loadings on the remaining subgroup factors fixed at zero. Consistent with the bifactor specification, correlations between the general factor and the subgroup factors were fixed at zero. Among the subgroup factors, only the correlation between Subgroup Factors 2 and 3 was freely estimated, while correlations involving Subgroup Factor 1 were fixed at zero. Each latent factor’s total variance was fixed at 1.0. Standardized loadings are reported. An asterisk denotes a statistically significant loading (Wald test, p < .05). Abbreviations: EFA = exploratory factor analysis; CFA = confirmatory factor analysis; ELIA = Emotion-Linked Interoceptive Awareness. | | | | | | | | | | | |

Supplementary Table 2 reports CFA fit indices for the bifactor model and three alternative models: unidimensional, correlated-factor, and second-order. The unidimensional model posited a single latent factor across 39 items. The correlated-factor model specified three latent factors that could correlate. Consistent with the revised bifactor model described in the main manuscript, only the correlation between the Anxiety and Irritation factors was freely estimated, and the correlations with the Positive Emotions factor were constrained to zero. Item-factor assignments followed the EFA results. The second-order model added a single higher-order factor to the correlated-factor model. In contrast, the bifactor model added a single general factor underlying all 39 items, which was specified to be independent of the three subgroup factors. Across indices, the bifactor model fit best; it was the only model to reach the prespecified acceptability thresholds and yielded the smallest Akaike Information Criterion (AIC) among the four models.

| **Supplementary Table 2.** Comparison of CFA model fit measures for ELIA | | | | | |
| --- | --- | --- | --- | --- | --- |
| Model | χ^2^/df | CFI | RMSEA | SRMR | AIC |
| Unidimensional | 9.873 | .618 | .142 | .147 | 45074.29 |
| Correlated-factor | 4.095 | .882 | .079 | .239 | 41015.94 |
| Second-order | 3.899 | .891 | .076 | .045 | 40875.08 |
| Bifactor | 3.334 | .919 | .067 | .037 | 40430.46 |
| Note. Model parameters were estimated using robust maximum likelihood. N = 415. Abbreviations: CFA = confirmatory factor analysis; ELIA = Emotion-Linked Interoceptive Awareness; CFI = comparative fit index; RMSEA = root mean square error of approximation; SRMR = standardized root mean square residual; AIC = Akaike Information Criterion. | | | | | |

1. **Sensitivity Analysis: Removing the Positive Emotions Subgroup Factor (Study 2)**

In the bifactor model specified in the main analyses, the Positive Emotions subgroup factor accounted for minimal unique variance beyond that explained by the general factor. To examine whether this subgroup factor was necessary, a sensitivity analysis was conducted by fitting an alternative model wherein the Positive Emotions items loaded on the general factor only. This alternative model evaluates the robustness of the substantive conclusions to omitting the Positive Emotions-specific subgroup factor without changing the remainder of the bifactor structure. Robustness was evaluated by comparing global model fit, bifactor indices, and the standardized general-factor loadings of the Positive Emotions items across the primary and alternative models (Supplementary Table 3). Overall, model fit and bifactor indices were broadly comparable across models; however, the CFI for the alternative model decreased marginally below the prespecified acceptance criterion utilized in the main text (CFI > .90; Hu and Bentler, 1999). Collectively, these results suggest that the substantive conclusions are largely robust to omitting the Positive Emotions-specific subgroup factor, though the overall model fit is numerically poorer when this subgroup factor is removed.

| **Supplementary Table 3.** Model comparisons: primary bifactor model versus alternative model without the Positive Emotions subgroup factor (global fit indices, bifactor indices, and Positive Emotions items’ general-factor loadings). | | | |
| --- | --- | --- | --- |
|  |  | Primary | Alternative |
| **Global fit indices** |  |  |  |
| χ^2^/df |  | 3.334 | 3.941 |
| CFI |  | .919 | .891 |
| RMSEA |  | .067 | .077 |
| SRMR |  | .037 | .042 |
| AIC |  | 40430.46 | 40874.98 |
| **Bifactor indices** |  |  |  |
| ECV |  | .65 | .67 |
| ωH |  | .88 | .88 |
| ωHS _Anxiety_ |  | .69 | .69 |
| ωHS _Irritation_ |  | .70 | .70 |
| **General-factor loadings** |  |  |  |
| **When I feel happy, I notice** |  |  |  |
| my body temperature rising and warmth spreading through my whole body. |  | .80 | .80 |
| my heart rate increasing and my heart pounding in my chest. |  | .73 | .74 |
| my body feeling light. |  | .84 | .85 |
| my breathing becoming easier. |  | .81 | .82 |
| **When I feel excited, I notice** |  |  |  |
| my body temperature rising and warmth spreading through my whole body. |  | .80 | .81 |
| my body feeling light. |  | .76 | .76 |
| my eyes opening wide. |  | .74 | .74 |
| a spring in my step. |  | .76 | .77 |
| **When I feel relaxed, I notice** |  |  |  |
| my body feeling light. |  | .80 | .79 |
| my breathing becoming slower, deeper, and easier. |  | .79 | .77 |
| my heartbeat slowing and my heart rate becoming steady. |  | .79 | .78 |
| my head feeling clear. |  | .75 | .74 |
| my muscles releasing tension. |  | .75 | .74 |
| **When I feel content, I notice** |  |  |  |
| the tension leaving my whole body. |  | .80 | .81 |
| my body feeling light. |  | .85 | .85 |
| warmth spreading through my whole body. |  | .83 | .83 |
| my breathing becoming slower, deeper, and easier. |  | .88 | .88 |
| my heartbeat slowing. |  | .85 | .85 |
| my face relaxing into a natural smile. |  | .79 | .79 |
| my head feeling clear. |  | .83 | .83 |
| my chest feeling full. |  | .84 | .84 |
| a comfortable fullness in my stomach. |  | .70 | .70 |
| Note. Model fit was evaluated using CFA. Model parameters were estimated using robust maximum likelihood. N = 415. The primary model was a bifactor CFA wherein all 39 ELIA items loaded on a general factor and, simultaneously, on their respective emotion-domain subgroup factors (Positive Emotions, Anxiety, and Irritation). The alternative model was identical to the primary bifactor specification, except that the Positive Emotions items loaded on the general factor only (i.e., the Positive Emotions-specific subgroup factor was omitted), with the remainder of the bifactor structure remaining unchanged. Models were compared in terms of global fit indices, bifactor indices, and the standardized general-factor loadings of the Positive Emotions items. Bifactor indices encompassed ECV, which indexes the proportion of common variance attributable to the general factor; ωH, which estimates the general factor score’s reliability; and ωHS, which estimates each subgroup factor’s reliability after accounting for the general factor. Abbreviations: CFA = confirmatory factor analysis; ELIA = Emotion-Linked Interoceptive Awareness; ECV = explained common variance; CFI = comparative fit index; RMSEA = root mean square error of approximation; SRMR = standardized root mean square residual; AIC = Akaike Information Criterion. | | | |

1. **Practice Types and Duration among Mind-Body Practice Practitioners (Study 4)**

Study 4 examined whether long-term engagement in mind-body practices (MBP) is associated with ELIA scores. Individuals who had engaged in MBPs for at least five years were classified as practitioners (n = 200), and those who had no MBP experience were classified as non-practitioners (n = 600), yielding a total sample of 800 participants. Owing to space constraints in the main manuscript, details about which MBPs practitioners engaged in and their durations were omitted. This section provides these details. Supplementary Table 4 reports the mean age and gender distribution of the practitioner and non-practitioner groups and, for the practitioner group, the type(s) of MBP and duration of practice. Forty-three inattentive respondents (who failed the trap item) were excluded from this table.

| **Supplementary Table 4.** Demographic characteristics and MBP profiles: practitioners vs. non-practitioners. | | |
| --- | --- | --- |
|  | Practitioners | Non-practitioners |
| N | 168 | 589 |
| Age (mean (SD)) | 50.36 (11.62) | 49.23 (12.17) |
| Gender = Woman (%) | 102 (60.7) | 248 (42.1) |
| Type of training (%) |  |  |
| Autogenic training | 8 (4.8) | 0 (0) |
| Body scan meditation | 3 (1.8) | 0 (0) |
| Breathing meditation | 14 (8.3) | 0 (0) |
| Mindfulness | 10 (6.0) | 0 (0) |
| Pilates | 10 (6.0) | 0 (0) |
| Tai chi | 8 (4.8) | 0 (0) |
| Yoga | 105 (62.5) | 0 (0) |
| Zazen | 10 (6.0) | 0 (0) |
| Years of continued training (%) |  |  |
| ≥10 years | 86 (51.2) | 0 (0) |
| ≥5 years | 82 (48.8) | 0 (0) |
| Note. Abbreviations: MBP = mind-body practice | | |

1. **Matching Diagnostics and Outcomes for the Mind-Body Practice Practitioner and Non-Practitioner Groups (Study 4)**

Study 4 included 168 MBP practitioners and 589 non-practitioners. To reduce size imbalance while aligning age and gender across groups, an age- and gender-matched subset of non-practitioners was created using propensity score matching. The propensity score, defined as the probability of being an MBP practitioner given age and gender, was estimated with logistic regression; groups were then matched on this score to obtain a control subset comparable in age and gender. Matching used 1:1 nearest-neighbor matching without replacement, and unmatched controls were excluded. Analyses were performed in R using the MatchIt package (Ho et al., 2011). After matching, the standardized mean differences (SMDs) for age and gender were both < 0.10, a commonly used threshold indicating negligible imbalance (Austin, 2011). Supplementary Figure 2 depicts kernel density plots for age and bar plots for gender in the full sample (“Original”) versus the matched sample (“Matched”); the age distributions show greater overlap after matching, and the gender proportions are aligned by design, illustrating improved alignment between groups.


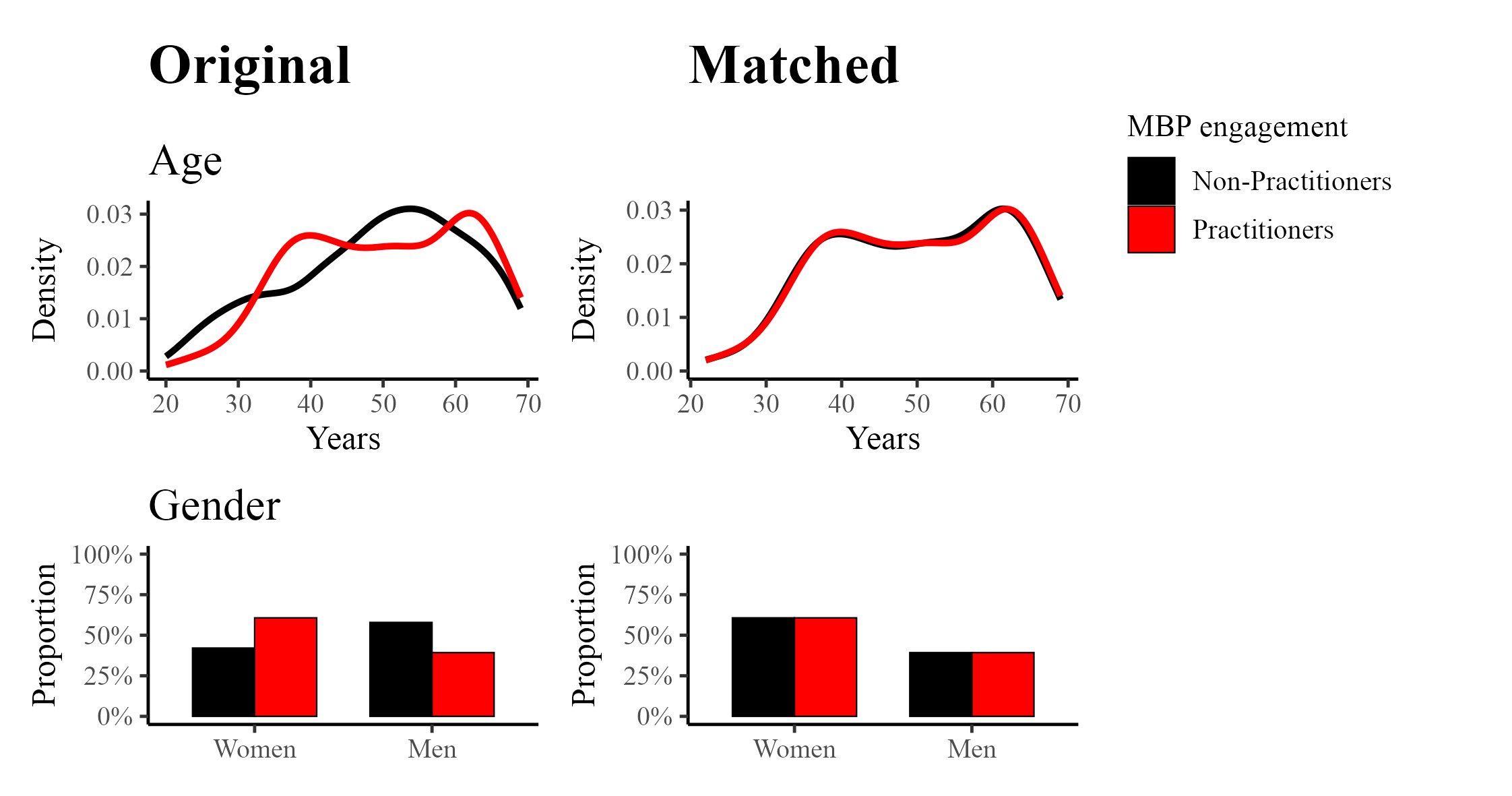


**Supplementary Figure 2.** Covariate balance on age and gender before and after propensity-score matching.

The propensity score (the probability of being an MBP practitioner conditional on age and gender) was estimated using logistic regression. The original sample comprised 168 practitioners and 589 non-practitioners; finally, 168 matched controls were selected. After matching, standardized mean differences for both covariates were < 0.10, indicating negligible residual imbalance. Abbreviation: MBP = mind-body practice.

1. **95% Confidence Intervals for ELIA Correlations with Other Self-Report Measures (Study 4)**

Study 4 examined whether the ELIA scales were correlated with (a) existing measures of interoceptive sensibility and (b) psychological traits that have been associated with interoception (trait anxiety and alexithymia). In addition to the ELIA, participants completed the Multidimensional Assessment of Interoceptive Awareness (MAIA; Shoji et al., 2018), Body Perception Questionnaire–Body Awareness subscale (BPQ-BA; Kobayashi et al., 2021), State-Trait Anxiety Inventory–Trait Version (STAI-T; Hidano et al., 2000), and Toronto Alexithymia Scale (TAS-20; Komaki et al., 2003). Owing to space constraints, the main text reports only the correlation coefficients and their statistical significance based on tests of the null hypothesis of zero correlation; it does not present 95% confidence intervals. Therefore, Supplementary Table 5 provides the 95% confidence intervals for all reported correlations.

| **Supplementary Table 5.** Confidence intervals for Study 4 correlations (ELIA, MAIA, BPQ-BA, STAI-T, and TAS-20). | | | | | | | | | | | | |
| --- | --- | --- | --- | --- | --- | --- | --- | --- | --- | --- | --- | --- |
|  | Et | Es1 | Es2 | Es3 | Mt | Ms1 | Ms2 | Ms3 | Ms4 | Ms5 | Ms6 | B |
| Mt | [.49,  .61] | [.55,  .66] | [.23,  .38] | [.24,  .38] | — | — | — | — | — | — | — | — |
| Ms1 | [.40,  .52] | [.49,  .60] | [.12,  .27] | [.14,  .29] | — | — | — | — | — | — | — | — |
| Ms2 | [.46,  .57] | [.53,  .63] | [.20,  .35] | [.19,  .34] | — | — | — | — | — | — | — | — |
| Ms3 | [.49,  .60] | [.47,  .58] | [.33,  .46] | [.34,  .48] | — | — | — | — | — | — | — | — |
| Ms4 | [.51,  .62] | [.58,  .68] | [.22,  .37] | [.22,  .37] | — | — | — | — | — | — | — | — |
| Ms5 | [.39,  .52] | [.51,  .62] | [.07,  .23] | [.11,  .27] | — | — | — | — | — | — | — | — |
| Ms6 | [–.46,  –.33] | [–.42,  –.28] | [–.42,  –.28] | [–.38,  –.23] | — | — | — | — | — | — | — | — |
| B | [.32,  .46] | [.16,  .31] | [.44,  .56] | [.37,  .50] | [.09,  .25] | [.01,  .17] | [.04,  .20] | [.19,  .34] | [.07,  .23] | [–.01,  .15] | [–.35,  –.20] | — |
| S | [–.08,  .09] | [–.29,  –.14] | [.24,  .38] | [.19,  .34] | [–.36,  –.22] | [–.44,  –.30] | [–.37,  –.22] | [–.20,  –.05] | [–.35,  –.20] | [–.51,  –.38] | [–.16,  –.00] | [.25,  .39] |
| Tt | [.02,  .18] | [–.14,  .02] | [.25,  .40] | [.16,  .31] | [–.21,  –.05] | [–.29,  –.14] | [–.19,  –.03] | [–.10,  .06] | [–.18,  –.02] | [–.35,  –.20] | [–.24,  –.08] | [.24,  .39] |
| Ts1 | [.18,  .33] | [.01,  .17] | [.36,  .49] | [.26,  .41] | [–.07,  .10] | [–.16,  –.00] | [–.04,  .12] | [.02,  .18] | [–.04,  .12] | [–.22,  –.06] | [–.29,  –.13] | [.30,  .44] |
| Ts2 | [–.01,  .15] | [–.16,  –.00] | [.19,  .34] | [.14,  .30] | [–.19,  –.03] | [–.27,  –.11] | [–.22,  –.06] | [–.08,  .08] | [–.19,  –.03] | [–.32,  –.17] | [–.28,  –.12] | [.20,  .35] |
| Ts3 | [–.29,  –.13] | [–.32,  –.16] | [–.17,  –.00] | [–.21,  –.05] | [–.34,  –.19] | [–.35,  –.20] | [–.30,  –.15] | [–.31,  –.15] | [–.30,  –.14] | [–.36,  –.21] | [.03,  .19] | [–.10,  .06] |
| Note. N = 589. Cells report 95% CIs for Pearson’s r. Only the lower triangle is presented (diagonal omitted). Rows for ELIA variables (Et, Es1–Es3) are omitted because intercorrelations among ELIA scales were not a primary focus; for the same reason, within-MAIA correlations (i.e., between MAIA Total and its subscales, and among MAIA subscales) are also omitted. Abbreviations: CI = confidence interval; ELIA = Emotion-Linked Interoceptive Awareness; MAIA = Multidimensional Assessment of Interoceptive Awareness; BPQ-BA = Body Perception Questionnaire–Body Awareness; STAI-T = State–Trait Anxiety Inventory, Trait; TAS-20 = 20-item Toronto Alexithymia Scale. Row and column labels use the following abbreviations: ELIA (Et = Total; Es1 = Positive Emotions; Es2 = Anxiety; Es3 = Irritation); MAIA (Mt = Total; Ms1 = Attention Regulation; Ms2 = Body Listening; Ms3 = Noticing; Ms4 = Emotional Awareness; Ms5 = Trusting; Ms6 = Not-Distracting); B = BPQ-BA; S = STAI-T; TAS-20 (Tt = Total; Ts1 = Difficulty Identifying Feelings; Ts2 = Difficulty Describing Feelings; Ts3 = Externally Oriented Thinking). | | | | | | | | | | | | |

1. **Mind-Body Practice Practitioners versus Non-Practitioners: Group Comparison of Multidimensional Assessment of Interoceptive Awareness and Body Perception Questionnaire**–**Body Awareness Subscale Scores (Study 4)**

Study 4 examined whether the mean ELIA scores differed between long-term MBP practitioners and age- and gender-matched non-practitioners. As the main manuscript primarily focused on evaluating whether long-term MBP engagement was associated with ELIA scores, group differences in the MAIA and BPQ-BA were not presented. For completeness, we report independent-samples t-tests comparing MAIA and BPQ-BA scores between MBP practitioners and their matched controls, while p-values are not corrected for multiple comparisons. Supplementary Table 6 presents the t-test results for the MAIA Total and subscale scores, as well as the BPQ-BA scores. Except on MAIA Subscale 6 (Not-distracting), practitioners scored significantly higher than non-practitioners.

| **Supplementary Table 6.** Group comparison of MAIA and BPQ-BA scores: MBP practitioners versus matched controls. | | | | | | | |
| --- | --- | --- | --- | --- | --- | --- | --- |
|  | MBP practitioners |  | Matched controls |  |  |  |  |
|  | M (SD) |  | M (SD) | t | df | p | d |
| MAIA Total | 2.70 (1.01) |  | 2.17 (0.95) | 4.96 | 332.79 | <.001 | .542 |
| MAIA Subscale 1 | 2.77 (1.07) |  | 2.24 (1.07) | 4.50 | 334.00 | <.001 | .491 |
| MAIA Subscale 2 | 2.67 (1.22) |  | 2.00 (1.09) | 5.34 | 329.68 | <.001 | .583 |
| MAIA Subscale 3 | 2.67 (1.10) |  | 2.16 (1.01) | 4.46 | 331.24 | <.001 | .486 |
| MAIA Subscale 4 | 2.81 (1.28) |  | 1.98 (1.21) | 6.08 | 332.90 | <.001 | .664 |
| MAIA Subscale 5 | 2.88 (1.20) |  | 2.34 (1.15) | 4.21 | 333.29 | <.001 | .459 |
| MAIA Subscale 6 | 2.68 (0.98) |  | 2.74 (0.98) | 0.59 | 334.00 | .554 | .065 |
| BPQ-BA | 1.96 (0.75) |  | 1.74 (0.57) | 3.01 | 310.66 | .003 | .328 |
| Note. Both groups N = 168. Tests are Welch’s t-tests (two-sided). p-values are uncorrected for multiple comparisons. MAIA Total represents the average of all 25 items. Abbreviations: MAIA = Multidimensional Assessment of Interoceptive Awareness; BPQ-BA = Body Perception Questionnaire–Body Awareness subscale; MBP = mind-body practice; M = mean; SD = standard deviation. MAIA Subscale labels: Subscale 1 = Attention regulation; Subscale 2 = Body listening; Subscale 3 = Noticing; Subscale 4 = Emotional awareness; Subscale 5 = Trusting; Subscale 6 = Not-distracting. | | | | | | | |

1. **Complete Results of the Mediation Analysis (Study 4)**

Study 4 examined whether ELIA scores mediated the association between long-term engagement in MBPs and psychological traits (namely, trait anxiety and alexithymia). Mediation analyses were specified to evaluate the ELIA total score and three ELIA subscale scores as potential mediators, and the STAI-T as well as the TAS-20 total score and its three TAS-20 subscale scores as potential outcomes. As a prerequisite step for mediation testing, group mean differences between long-term MBP practitioners and non-practitioners were examined for these variables. Subsequently, mediation analyses were conducted only for ELIA indices and psychological-trait variables that exhibited significant group differences.

Based on this selection step, the mediation analyses reported in the main text focused on the ELIA total score and ELIA Subscale 1 score as mediators, and on the STAI-T and TAS-20 Subscale 3 score (Externally Oriented Thinking) as outcomes. In the mediation models, long-term MBP engagement was the predictor, coded as 0 (non-practitioner) and 1 (practitioner). Models were estimated using structural equation modeling. Indirect effects were evaluated using 95% bias-corrected and accelerated bootstrap confidence intervals.

The main manuscript reports only mediation models that yielded statistically significant indirect effects. For completeness, Supplementary Figure 3 (Panel A) presents a mediation model with a non-significant indirect effect. Additionally, although ELIA Subscales 2 and 3 did not exhibit significant mean differences between long-term MBP practitioners and non-practitioners, the corresponding mediation models are also presented in Supplementary Figure 3 (Panels B–E).


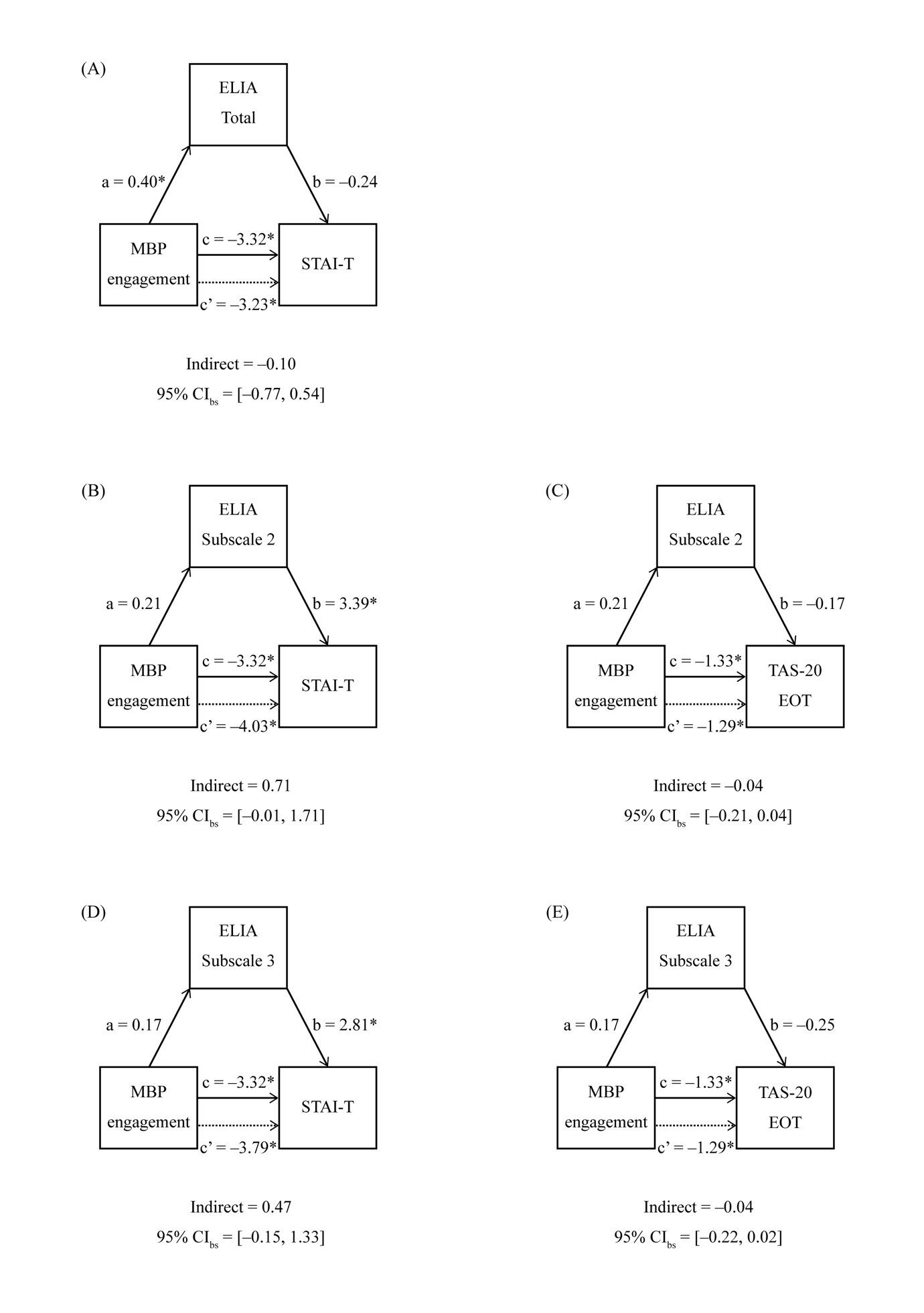


**Supplementary Figure 3.** No significant ELIA-mediated associations between MBP engagement and STAI-T/TAS-20 EOT.

Panels A–E depict mediation models assessing whether ELIA scores mediate the association between MBP engagement and psychological traits: (A) MBP engagement → ELIA Total → STAI-T; (B) MBP engagement → ELIA Subscale 2 → STAI-T; (C) MBP engagement → ELIA Subscale 2 → TAS-20 EOT; (D) MBP engagement → ELIA Subscale 3 → STAI-T; and (E) MBP engagement → ELIA Subscale 3 → TAS-20 EOT. Path notation: a (MBP engagement → ELIA), b (ELIA → trait), c′ (direct effect: MBP engagement → trait), and c (total effect: direct + indirect = c′ + a×b). Arrow labels indicate standardized path coefficients with 95% bias-corrected and accelerated (BCa) bootstrap CIs based on 5,000 resamples. The indirect effect (a×b) and its 95% BCa bootstrap CI are reported below each panel. Statistical significance for all displayed effects was determined by whether the 95% BCa bootstrap CI excludes zero; asterisks denote effects meeting this criterion. Solid lines indicate significant effects, while dashed lines indicate non-significant effects according to this CI-based criterion. N = 336. ELIA Total is the mean of 39 items; Subscale 2 indexes bodily awareness during anxiety, and Subscale 3 indexes bodily awareness during irritation. MBP engagement was coded 0 = non-practitioner and 1 = practitioner. Abbreviations: MBP = mind-body practice; ELIA = Emotion-Linked Interoceptive Awareness; STAI-T = State–Trait Anxiety Inventory (Trait); TAS-20 = 20-item Toronto Alexithymia Scale; EOT = Externally Oriented Thinking (TAS-20 subscale); CI = confidence interval.

7. **Supplementary References**

Austin, P. C. (2011). An introduction to propensity score methods for reducing the effects of confounding in observational studies. Multivariate Behav. Res. 46, 399-424. doi: 10.1080/00273171.2011.568786.

Clauset, A., Newman, M. E., and Moore, C. (2004). Finding community structure in very large networks. Phys. Rev. E. Stat. Nonlin. Soft. Matter. Phys. 70, 066111. doi: 10.1103/PhysRevE.70.066111.

Hagberg, A. A., Schult, D. A., and Swart, P. J. (2008). Exploring network structure, dynamics, and function using NetworkX. In: Varoquaux, G., Vaught, T., and Millman, J. (eds.), Proceedings of the 7th Python in Science Conference (SciPy 2008), 11–15. doi: 10.25080/TCWV9851.

Hidano, N., Fukuhara, M., Iwawaki, M., Soga, S., and Spielberger, C. D. (2000). State–Trait Anxiety Inventory (Form JYZ) manual. Jitsumukyoiku Shuppan.

Ho, D., Imai, K., King, G., and Stuart, E. A. (2011). MatchIt: Nonparametric preprocessing for parametric causal inference. J. Stat. Softw. 42, 1-28. doi: 10.18637/jss.v042.i08.

Honnibal, M., Montani, I., Van Landeghem, S., and Boyd, A. (2023). spaCy (Version 3.7.2) [Computer software]. Explosion. doi: 10.5281/zenodo.1212303.

Hu, L. T., and Bentler, P. M. (1999). Cutoff criteria for fit indexes in covariance structure analysis: Conventional criteria versus new alternatives. Struct. Equ. Model. 6, 1-55. doi: 10.1080/10705519909540118.

Ikegami, Y. (2025). neologdn (Version 0.5.4) [Computer software]. https://github.com/ikegami-yukino/neologdn

Kobayashi, R., Honda, T., Machizawa, M., Ichikawa, N., and Nakao, T. (2021). Factor structure, reliability and validation of the Japanese version of the body perception questionnaire-body awareness very short form (BPQ-BAVSF-J). Jpn. J. Res. Emot. 28, 38-48. doi: 10.4092/jsre.28.2_38.

Komaki, G., Maeda, M., Arimura, T., Nakata, A., Shinoda, H., Ogata, I. et al. (2003). The reliability and factorial validity of the Japanese version of the 20-item Toronto Alexithymia Scale (TAS-20). Jpn. Psychosom. Med. 43, 839-846. doi: 10.15064/jjpm.43.12_839.

Megagon Labs. (2024). GiNZA (Version 5.2.0) [Computer software]. https://pypi.org/project/ginza/

Shoji, M., Mehling, W. E., Hautzinger, M., and Herbert, B. M. (2018). Investigating multidimensional interoceptive awareness in a Japanese population: validation of the Japanese MAIA-J. Front. Psychol. 9, 1855. doi: 10.3389/fpsyg.2018.01855.
