# Supplementary figures and images for: Understanding interoception across emotional contexts: development and validation of the Emotion-Linked Interoceptive Awareness Scale
Source: Front Psychol. 2026 Mar 18;17:1757948. doi: 10.3389/fpsyg.2026.1757948 (PMC13040479; doi:10.3389/fpsyg.2026.1757948)

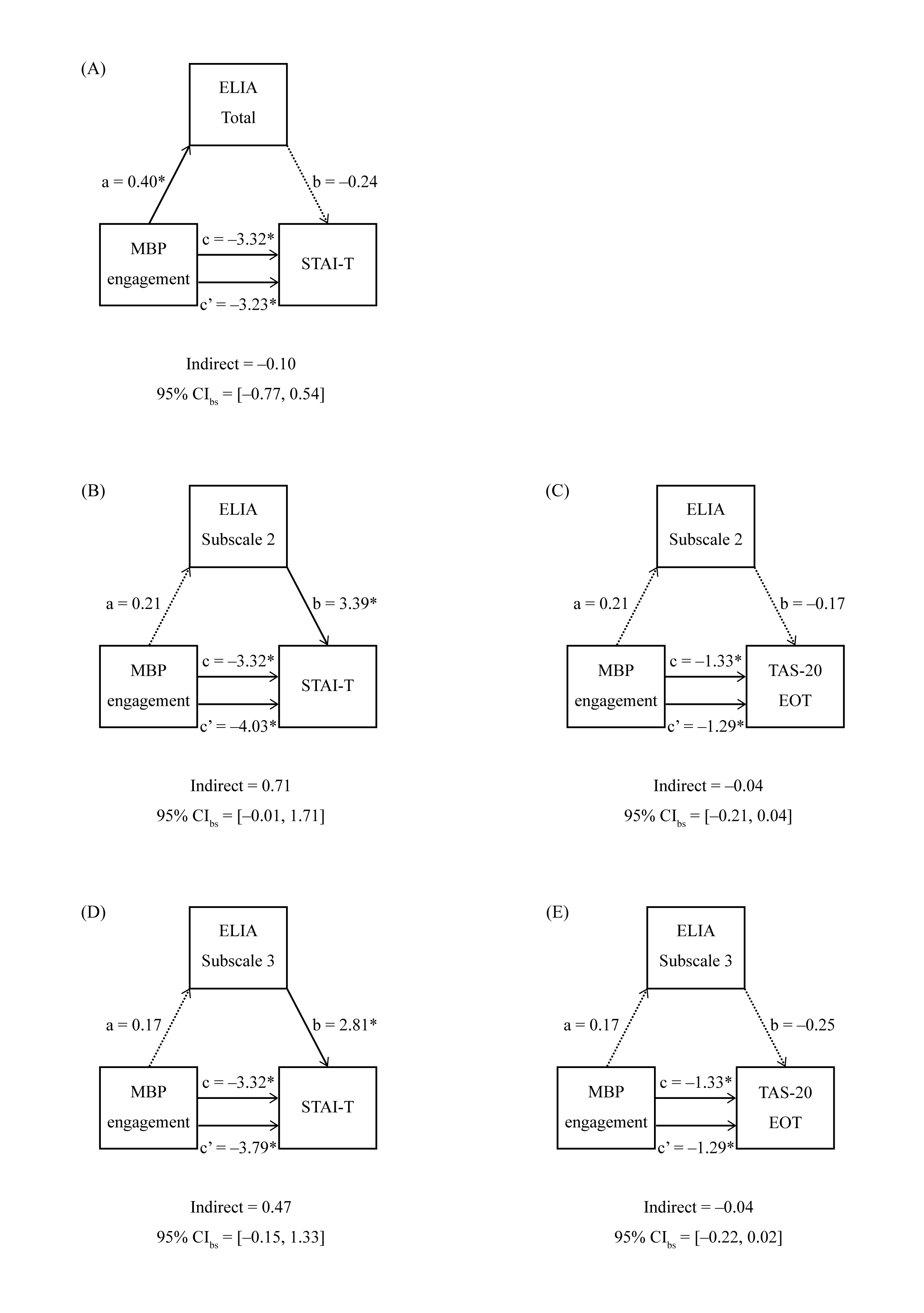

Supplement: Supplementary file 2 [file Image_1.tif]
